# Phase-specific neuromechanical signatures of 40-m sprint performance: an integrated two-dimensional kinematic and surface electromyographic profiling study

**DOI:** 10.3389/fphys.2026.1939386

**Published:** 2026-10-09

**Authors:** Xin Wang

**Affiliations:** School of Physical Education, Qilu Normal University, Jinan, China

**Keywords:** excitation movement coupling, neuromechanical signature, phase-specific profiling, sprint biomechanics, surface electromyography

## Abstract

**Background:**

Sprint performance reflects coordinated mechanical and neuromuscular behavior across distinct sprint phases. This study identified phase-specific neuromechanical signatures of 40-m sprint performance and tested whether integrated kinematic and electromyographic information added predictive value beyond direct spatiotemporal determinants of running velocity.

**Methods:**

In this cross-sectional study, 160 young adults classified as sprint-trained athletes or physically active non-sprinters completed three maximal 40-m sprints. Split times were recorded every 10 m. Four synchronized high-speed cameras (240 frames·s^-1)^ and wireless surface electromyography of six lower-limb muscles captured sagittal-plane kinematics, muscle excitation, coactivation, and excitation–movement coupling across four sprint phases. Velocity-adjusted mixed-effects models and one-dimensional statistical parametric mapping assessed discrete and waveform differences. Reliability-qualified features were analyzed using elastic-net models with repeated nested five-fold cross-validation. Mean 40-m velocity was the primary outcome. Step length and step frequency formed the direct spatiotemporal benchmark; ground-contact and flight times were added in an extended benchmark. Sprint-training-status classification was secondary and exploratory.

**Results:**

Sprint-trained athletes completed 40 m faster than non-sprinters (5.20 ± 0.31 vs 5.95 ± 0.40 s). Their profile showed longer normalized steps, shorter contact times, phase-specific trunk and lower-limb configurations, greater early-phase gluteus maximus and vastus lateralis excitation, and greater later-phase biceps femoris preactivation and medial gastrocnemius excitation. In the complete synchronized sample ( n=152), the integrated non-spatiotemporal model achieved $R^{2}=0.74$and RMSE = 0.34 m·s^-1^, versus $R^{2}=0.60$/RMSE = 0.43 for kinematics and $R^{2}=0.55$/RMSE = 0.45 for sEMG. Adding neuromechanical features to the direct benchmark improved *R*^2^ from 0.72 to 0.86 and reduced RMSE from 0.36 to 0.25 m·s^-1^ (paired reduction 0.11, 95% CI 0.07–0.15). Improvement persisted for the extended benchmark (*R*^2^ 0.76→0.87; RMSE 0.33→0.24). Transition and high-speed features accounted for 82% of coefficient magnitude, not variance explained. Exploratory classification achieved AUC = 0.94 and balanced accuracy = 0.89.

**Conclusion:**

Forty-metre sprint performance was characterized by a phase-specific neuromechanical signature that provided incremental out-of-fold predictive information beyond direct and closely related spatiotemporal velocity metrics. External validation is required before broader generalization.

## Introduction

Sprint performance is a decisive determinant of success in track-and-field events and in many team sports in which athletes must accelerate rapidly, attain high running velocity, and execute high-intensity locomotor actions under severe temporal constraints (Burgomaster et al., 2014). Short-distance sprint testing is therefore widely used to evaluate speed-related capability, monitor adaptation to training, and support athlete development. The 40-m sprint is particularly informative because it encompasses the progression from initial acceleration through continued acceleration and transition toward high-speed running, thereby exposing athletes to distinct mechanical and neuromuscular demands within a single performance task (Deliceoglu et al., 2024). Sprint performance, however, cannot be adequately represented by completion time alone. It is an emergent neuromechanical outcome arising from the coordinated interaction of step organization, segmental positioning, temporal control, and phase-specific muscle excitation (Janowski et al., 2017).

Most sprint-biomechanics research has traditionally examined discrete performance determinants, including step length, step frequency, contact time, flight time, lower-limb joint angles, and trunk orientation (Valamatos et al., 2022). These variables have substantially advanced understanding of sprint mechanics by showing that faster running is associated with characteristic spatiotemporal and postural features (Kakehata et al., 2020). Nevertheless, the explanatory value of any single variable is inherently limited. Sprint velocity emerges from the combined expression of multiple movement characteristics, and similar performance outcomes may be achieved through different configurations of stride mechanics and segmental organization (King et al., 2023). Importantly, step length and step frequency are not merely correlates of running velocity but direct mathematical constituents of it, because running velocity is determined by their product; contact and flight times are likewise closely related to the temporal organization underlying step frequency. Consequently, predictive models that include these variables may partly reconstruct velocity from its underlying spatiotemporal components rather than identify independent neuromechanical information. A longer step, for example, may contribute positively to velocity only when it is accompanied by an appropriate step frequency and sufficiently brief ground-contact duration (Maćkała and Mero, 2013). Accordingly, interpreting kinematic variables in isolation may overlook the compensatory and coordinated relationships through which sprint performance is generated.

This limitation has encouraged a transition from single-variable interpretation toward multidimensional profiling. Within this framework, a neuromechanical signature can be conceptualized as a reproducible and interpretable multivariable configuration of movement and muscle-excitation characteristics that collectively characterizes sprint performance (Lockie et al., 2015). Such a signature should not be regarded as a universal ideal technique. Rather, it reflects the coordinated strategy through which an athlete satisfies the changing temporal and mechanical constraints of sprinting (Martín-Fuentes and van den Tillaar, 2022). In the present context, a neuromechanical signature is operationally defined as a cross-validated pattern of kinematic and electromyographic features that jointly characterizes and predicts sprint performance and differentiates sprint-training backgrounds more effectively than any individual variable (Mattes et al., 2021). This distinction is important because sprint proficiency is unlikely to depend on a single optimal characteristic; it is more plausibly represented by the coordinated organization of several phase-dependent movement and neuromuscular features (Clark et al., 2025; Meng et al., 2026).

A purely kinematic approach cannot fully explain the mechanisms underlying visible sprint technique (Maćkała and Mero, 2013; Mero et al., 1992). Surface electromyography provides complementary information regarding the temporal organization and relative amplitude of muscle excitation and may therefore help clarify how observed movement patterns are supported by neuromuscular control (Morin et al., 2019; Morin et al., 2015). Differences in contact duration, joint configuration, or trunk inclination may be accompanied by distinct patterns of pre-activation before ground contact, excitation during stance, and intermuscular coordination across the stride cycle (Lockie et al., 2015; Murphy et al., 2003). These neuromuscular characteristics are particularly relevant in sprinting because the time available to prepare for and respond to ground contact becomes progressively constrained as running velocity increases (Myrvang and Tillaar, 2024). Although surface electromyography does not directly quantify muscle force, joint torque, or motor-unit recruitment, its integration with kinematic analysis provides a more mechanistically informative representation of sprint performance than either modality considered independently.

The need for phase-specific analysis is equally important. Sprint mechanics and muscle-excitation patterns change substantially across a 40-m sprint. Initial acceleration is typically characterized by marked forward trunk inclination, relatively longer ground-contact periods, and rapid step-to-step increases in velocity (Ruiter et al., 2022; Ruiter and Dieën, 2019). As acceleration continues, step length and step frequency are reorganized, contact duration decreases, and lower-limb segment positions progressively adapt to the increasing demand for rapid repositioning (Abdelkrim et al., 2007; Bezodis et al., 2019; Salo et al., 2011). During the transition toward high-speed running, the athlete adopts a more upright posture and must coordinate muscle excitation within increasingly brief temporal windows (Schubert et al., 2014). Consequently, a single analysis zone or one isolated stride cannot adequately represent the neuromechanical organization of an entire 40-m sprint. A phase-specific approach is required to determine whether performance-related features remain consistent or change across initial acceleration, continued acceleration, transition, and high-speed phases.

Despite substantial progress in sprint research, several important gaps remain. Previous studies have often focused on elite sprinters, highly trained samples, or laboratory-based comparisons between faster and slower performers (Standing and Maulder, 2017; Weyand et al., 2000). Many have also examined either kinematics or electromyography separately, thereby providing limited insight into the integrated neuromechanical organization of sprinting (Clark et al., 2025; Struzik et al., 2016; Tillaar, 2021). In addition, broad classifications such as “trained athletes” may combine participants from sports with markedly different exposure to maximal sprinting, strength–power development, and technical training. Such heterogeneity complicates interpretation because observed differences may reflect sport-specific characteristics associated with prior training exposure, running velocity, or general athletic experience rather than sprint-specific proficiency. A more rigorous comparison therefore requires clearly defined sprint-training backgrounds, such as competitive sprint-trained athletes and physically active non-sprinters, together with quantitative characterization of training exposure.

A further methodological challenge is that many kinematic and electromyographic variables are inherently velocity dependent. Faster running is naturally accompanied by shorter contact times, altered joint configurations, and changes in muscle-excitation timing. Therefore, between-group differences cannot automatically be interpreted as superior technique or training adaptation. To distinguish characteristics associated with sprint-training status from changes attributable primarily to running velocity, phase-specific velocity must be incorporated into the analytical framework. A related concern arises when sprint velocity itself is modelled as an outcome: direct spatiotemporal constituents of velocity should be distinguished from joint, segmental, electromyographic, and excitation–movement features that are not mathematically embedded in the outcome. This distinction allows the additional neuromechanical information associated with sprint performance to be evaluated without conflating prediction with mathematical reconstruction of velocity. This issue is central to the identification of meaningful neuromechanical signatures because a valid signature should capture coordinated characteristics that explain performance beyond the trivial observation that faster individuals move differently because they are faster.

The translation of these concepts into applied practice remains challenging. Three-dimensional motion capture, force platforms, and instrumented laboratory systems provide detailed biomechanical information, but their routine implementation is restricted by cost, technical complexity, spatial requirements, and limited portability. High-speed two-dimensional sagittal-plane video analysis offers a complementary field-based approach that prioritizes feasibility, scalability, and ecological relevance. When camera placement, calibration, temporal resolution, event identification, marker digitization, and measurement reliability are rigorously standardized, 2D analysis can provide interpretable information on step mechanics, trunk posture, and lower-limb joint configurations. Wireless surface electromyography can be recorded concurrently during overground sprinting, allowing movement and muscle excitation to be evaluated under realistic performance conditions. The value of this integrated approach lies not in replacing laboratory-based three-dimensional or kinetic assessment, but in developing a practical and reproducible framework that can connect neuromechanical research with routine athlete monitoring.

This translational need is becoming increasingly urgent as coaching practice moves toward data-informed decision-making. Practitioners require assessment methods that are not only reliable and interpretable, but also sufficiently accessible for repeated use in training environments. An integrated neuromechanical profile could move sprint assessment beyond classifying athletes as merely faster or slower by identifying which movement and muscle-excitation characteristics are associated with performance at different phases of the sprint. Such information may support individualized technical feedback, improve the prioritization of training targets, and help determine whether performance differences are related primarily to sprint-specific skill, neuromuscular organization, or velocity-dependent neuromechanical characteristics.

Accordingly, the novelty of the present study lies in the simultaneous integration of phase-specific two-dimensional kinematics and surface electromyography within a multivariable profiling framework. Rather than relying on isolated between-group comparisons, the study evaluates whether coordinated patterns of spatiotemporal organization, sagittal-plane segmental configuration, and lower-limb muscle excitation define distinct neuromechanical signatures of 40-m sprint performance. The primary modelling framework compares kinematic-only, electromyographic-only, and integrated neuromechanical models to determine whether combining movement and muscle-excitation information improves prediction of sprint performance beyond either data domain alone. Sprint-training-status classification was examined separately as a secondary exploratory analysis to determine whether the neuromechanical profiles also discriminated sprint-trained athletes from physically active non-sprinters. For performance modelling, direct spatiotemporal constituents of running velocity are considered separately from non-spatiotemporal kinematic, electromyographic, and excitation–movement features, permitting the incremental information provided by the neuromechanical profile to be evaluated beyond the mathematical components of velocity itself. By accounting for phase-specific running velocity and using clearly defined sprint-training backgrounds, this approach aims to distinguish performance-associated neuromechanical characteristics from differences associated primarily with between-group variation in running velocity.

Therefore, this study aimed to identify phase-specific neuromechanical signatures of 40-m sprint performance by integrating high-speed two-dimensional sagittal-plane kinematics with synchronized surface electromyography in sprint-trained athletes and physically active non-sprinters. It was hypothesized that higher sprint performance would be characterized by a distinct phase-dependent profile comprising a more favorable balance between normalized step length and step frequency, shorter contact duration, phase-appropriate trunk and lower-limb configurations, and systematic differences in lower-limb muscle pre-activation, stance-phase excitation, and intermuscular coordination. It was further hypothesized that these performance-associated neuromechanical characteristics would vary across the initial-acceleration, continued-acceleration, transition, and high-speed phases, and that an integrated kinematic–electromyographic model would predict sprint performance more accurately than kinematic-only or electromyographic-only models after accounting for phase-specific running velocity. For continuous sprint-performance modelling, we further hypothesized that integrated neuromechanical features would provide predictive information beyond that attributable to the direct spatiotemporal constituents of running velocity. As a secondary exploratory objective, we additionally examined whether kinematic, electromyographic, and integrated neuromechanical profiles could discriminate sprint-training status; no confirmatory hypothesis was specified for this classification analysis.

## Materials and methods

### Study design and analytical framework

This cross-sectional neuromechanical profiling study integrated high-speed two-dimensional sagittal-plane kinematics with synchronized surface electromyography during maximal 40-m overground sprinting. Sprint-trained track-and-field athletes and physically active non-sprinters were included as prespecified reference groups, while sprint performance was also examined continuously. The primary objective was to determine whether the integration of kinematic and muscle-excitation characteristics provided a more informative representation of sprint performance than either modality alone. The study therefore combined conventional phase-specific comparisons with multivariable profiling to identify interpretable combinations of mechanical, electromyographic, and excitation–movement coupling features.

The analytical framework comprised three sequential stages: evaluation of measurement reliability, examination of phase-specific neuromechanical differences, and internally validated signature modelling. The performance-related signature constituted the primary analytical target, whereas discrimination between sprint-trained athletes and physically active non-sprinters was treated as a secondary analysis. Because of the cross-sectional design, findings were interpreted as performance-related associations rather than causal adaptations to sprint training. Reporting followed the Strengthening the Reporting of Observational Studies in Epidemiology statement.

### Participants, group classification, sample size, and pretest characterization

Participants were recruited between 1 and 26 September 2025 from university athletics programs, affiliated regional sports clubs, and the general undergraduate population. A total of 184 individuals were initially approached for participation; 176 proceeded to formal eligibility screening, whereas eight did not proceed to screening. Ten screened individuals did not satisfy the prespecified eligibility criteria, leaving 166 eligible candidates. Six eligible individuals subsequently declined participation before testing, resulting in a final enrolled sample of 160 participants. The complete recruitment, enrolment, and modality-specific analytical flow is presented in **Supplementary Figure S1**.

The final sample comprised 160 undergraduate students aged 18–24 years, including 80 sprint-trained track-and-field athletes and 80 physically active non-sprinters. The sample included 80 men and 80 women, with equal sex distribution within each group. Participants were recruited from university athletics programs, affiliated regional sports clubs, and the general undergraduate population. Sprint-trained participants competed in the 100-m, 200-m, or sprint-hurdle events and had completed at least three years of structured sprint training. Eligibility additionally required at least three training sessions per week, regular maximal-velocity exposure during the preceding six months, and participation in sanctioned competition within the previous 12 months. Regular maximal-velocity exposure was defined as at least one weekly session containing sprint efforts performed at ≥95% of documented maximal velocity. Training status was verified using a standardized questionnaire, electronically timed training records where available, and coach confirmation. Athletes specializing in endurance, field, aquatic, or other non-sprint disciplines were not included.

Physically active non-sprinters completed at least 150 min of moderate-to-vigorous physical activity per week during the preceding six months but had no history of formal sprint coaching, track-sprint competition, or structured speed training during the previous two years. They were also required not to participate regularly in sports involving repeated maximal-speed sprinting. Participants were excluded if they had sustained a lower-limb or trunk injury during the preceding six months, undergone surgery likely to affect sprint mechanics, were receiving musculoskeletal rehabilitation, or had cardiovascular or neurological conditions that could compromise maximal sprinting. Medication affecting neuromuscular function and skin conditions preventing secure electrode placement were additional exclusion criteria.

An *a priori* sample-size calculation was performed using G*Power version 3.1 for a fixed-model multiple linear regression analysis (*R*^2^ deviation from zero). Assuming a moderate effect size ( $f^{2}=0.15$), ten prespecified predictor terms, a two-sided significance level of 0.05, and 90% statistical power, a minimum of 147 participants was required. The target was increased to 160 to maintain balanced group and sex representation, improve estimate precision, and accommodate potentially unusable recordings. This conventional calculation was intended to support the prespecified association framework and was not interpreted as a formal sample-size justification for the subsequent high-dimensional regularized models.

For the high-dimensional analyses, sample-size adequacy was instead considered in relation to effective model complexity and the safeguards used to limit overfitting. Although the primary non-spatiotemporal candidate pool contained 314 reliability-qualified phase-specific features, complete synchronized multimodal data were available for 152 participants, creating a setting in which the initial number of candidate predictors exceeded the number of observations. This feature-to-sample relationship motivated the use of elastic-net regularization rather than conventional unpenalized multivariable regression. Model complexity was progressively reduced through prespecified reliability screening and fold-specific correlation filtering, after which a median of 121 candidate predictors (range 112–131) entered elastic-net estimation in the fully integrated model. Regularization further reduced the effective model size to a median of 22 non-zero coefficients per internal-validation resample (IQR 18–26), and only nine features satisfied the final stability criterion of selection in at least 70% of resamples with a consistent coefficient direction.

To minimize optimism under this high-dimensional setting, standardization, correlation filtering, feature selection, and hyperparameter tuning were performed exclusively within the training data of repeated nested five-fold cross-validation, and model performance was estimated from held-out out-of-fold observations. The same outer folds were used across competing models, and feature stability and predictive performance were additionally examined under prespecified sensitivity analyses. Accordingly, these analyses were regarded as internally validated exploratory model-development analyses rather than externally or prospectively validated prediction models. Independent external validation remains necessary before the derived neuromechanical signature can be considered generalizable.

Participant characterization included age, sex, body height, body mass, body mass index, recorded-leg dominance, leg length, thigh length, and shank length. For standardized single-limb analysis, the recorded leg was selected *a priori* as the participant’s preferred leg for kicking a ball, and the same selection rule was applied to all participants before inspection of sprint-derived kinematic or sEMG outcomes. Analysis was restricted to one prespecified limb because the primary objective was to characterize phase-specific neuromechanical performance patterns rather than bilateral asymmetry, and retaining one limb per participant provided a consistent participant-level sampling unit while avoiding additional within-participant dependence that would arise from treating bilateral measurements as separate observations. The selected leg was used as a standardized recording side and was not assumed to represent a universally biomechanically dominant limb. Consequently, the present design does not permit inference regarding inter-limb asymmetry or side-specific neuromechanical differences.

Leg length was measured from the anterior superior iliac spine to the medial malleolus and used to normalize step length. Sprint-training exposure was characterized by training age, weekly training frequency, sprint-specific volume, maximal-velocity exposure, resistance- and plyometric-training frequency, competition level, and season-best performance. Because the sprint-trained and non-sprinter groups were recruited partly from different source populations, group membership could also be associated with unmeasured characteristics such as strength, body composition, habitual sport participation, or previous training history. Group comparisons were therefore interpreted as characteristics associated with sprint-training status rather than as causal adaptations produced by sprint training.

Participants were instructed to avoid strenuous exercise for 48 h, alcohol for 24 h, and caffeine or other stimulants for at least 3 h before testing. They maintained their habitual diet and hydration, obtained at least seven hours of sleep, and reported illness, pain, fatigue, or muscle soreness on arrival. Testing was postponed when these factors were considered likely to compromise maximal sprint performance. The protocol was approved by the ethics committee of the School of Physical Education, Qilu Normal University and was conducted in accordance with the Declaration of Helsinki. All participants provided written informed consent before participation.

### Experimental procedures, sprint protocol, and phase definition

Participants completed one familiarization session and one experimental session separated by 48–72 h. During familiarization, they practiced the standardized starting procedure and completed submaximal accelerations while wearing the anatomical markers, wireless sEMG sensors, and standardized footwear. The experimental session began with a standardized 15-min warm-up comprising low-intensity running, dynamic lower-limb mobility, sprint-specific drills, and three progressive 30-m accelerations at approximately 70%, 85%, and 95% of perceived maximal effort. After at least 3 min of recovery, participants performed three maximal 40-m sprints from a two-point standing start, with 5 min of recovery between trials. The front foot was positioned 0.5 m behind the first timing gate, and the preferred starting-leg position was maintained across trials. Participants initiated each sprint voluntarily and were instructed to accelerate maximally beyond the finish line. Total and split times were recorded using G4d dual-beam timing gates (Swift Performance, Australia) positioned at 0, 10, 20, 30, and 40 m. Mean velocity was calculated for the full sprint and each 10-m interval by dividing distance by elapsed time. Because participants started behind the first gate, the first phase represented the electronically timed 0–10-m interval rather than the complete distance travelled from the initial foot position.

The fastest valid trial was selected for the primary analysis. The mean of the two fastest trials was retained for sensitivity analysis when their total sprint times differed by no more than 2%. Four operational distance-based phases were defined: initial acceleration (0–10 m), continued acceleration (10–20 m), transition (20–30 m), and high-speed running (30–40 m). These intervals were treated as standardized analytical zones rather than identical physiological or mechanical states across participants. All participants wore the same model of lightweight, non-spiked track shoes supplied in the appropriate size. The same footwear was used during familiarization and experimental testing. Trials were performed in the same lane on a level synthetic track. Ambient temperature, wind speed, and track-surface condition were recorded, and testing was postponed under unsafe or materially different environmental conditions.

### Integrated kinematic and sEMG data acquisition

Sagittal-plane kinematics and muscle excitation were acquired concurrently during each sprint. Four Chronos 2.1-HD high-speed cameras (Kron Technologies Inc., Burnaby, BC, Canada) were positioned perpendicular to the running direction at 5, 15, 25, and 35 m. Cameras recorded at 240 frames·s^-^¹ with a resolution of 1920 × 1080 pixels and a shutter speed of 1/2000 s. Each camera was mounted 1.1 m above the track and approximately 10 m from the running lane. Camera distance, optical axis, zoom, focus, and field of view were standardized across sessions. The operational sprint phases, camera locations, analysis zones, and corresponding performance outcomes are summarized in **Table 1**. Each analysis zone was calibrated using a rigid 6 × 2-m two-dimensional reference frame positioned in the sagittal plane of motion. High-contrast markers were placed on the recorded side over the acromion, greater trochanter, lateral femoral epicondyle, lateral malleolus, calcaneus, and fifth metatarsal head. Anatomical landmarks were identified by the same trained assessor, and participants wore close-fitting clothing to minimize marker occlusion and movement relative to the underlying segments.

**Table 1:** Operational definition of sprint phases and phase-specific data-acquisition framework.

Sprint phase	Timed interval	Camera position	Timing-gate interval	Kinematic sample	Performance outcome
Initial acceleration	0–10 m	5 m	0–10 m	Complete recorded-leg stride centred within the calibrated field	Mean 0–10-m velocity
Continued acceleration	10–20 m	15 m	10–20 m	Complete recorded-leg stride centred within the calibrated field	Mean 10–20-m velocity
Transition	20–30 m	25 m	20–30 m	Complete recorded-leg stride centred within the calibrated field	Mean 20–30-m velocity
High-speed running	30–40 m	35 m	30–40 m	Complete recorded-leg stride centred within the calibrated field	Mean 30–40-m velocity

Muscle excitation was recorded using a wireless Ultium EMG system (Noraxon USA Inc., Scottsdale, AZ, USA) at 2000 Hz. Bipolar sensors were positioned over the gluteus maximus, biceps femoris long head, rectus femoris, vastus lateralis, medial gastrocnemius, and tibialis anterior of the recorded leg. These muscles represented the principal hip-extensor, knee-flexor, knee-extensor, plantar-flexor, and dorsiflexor functions contributing to sprinting. Electrode placement and orientation followed standardized anatomical recommendations. The skin was shaved when necessary, lightly abraded, and cleaned with alcohol. Sensors were secured using elastic adhesive material to minimize displacement and movement artefact. Muscle-specific maximal voluntary isometric contractions were recorded before sprint testing. Participants performed three 5-s contractions for each muscle, separated by 60 s of recovery, with 2 min between muscles. Testing order was randomized, standardized verbal encouragement was provided, and resistance was applied using non-elastic straps attached to a fixed frame.

The gluteus maximus was tested in prone lying with the knee flexed to 90° during resisted hip extension. The biceps femoris was tested in prone lying with the knee flexed to 45° during resisted knee flexion. The rectus femoris and vastus lateralis were tested while seated with the hip flexed to 90° and the knee at 60° of flexion during resisted knee extension. The medial gastrocnemius was tested in prone lying with the knee extended during resisted plantar flexion, whereas the tibialis anterior was tested while seated during resisted ankle dorsiflexion. Video and sEMG recordings were synchronized using a common transistor–transistor logic trigger. A single trigger generated a digital pulse within the sEMG recording and simultaneously activated four light-emitting diodes, with one diode positioned within each camera field of view. This provided a shared temporal reference between the continuous sEMG recording and the four video streams. The integrated experimental setup, including the timing-gate configuration, camera locations, calibrated analysis zones, anatomical landmarks, sEMG sensor placement, and synchronization framework, is illustrated in **Figure 1**.

**Figure 1:**
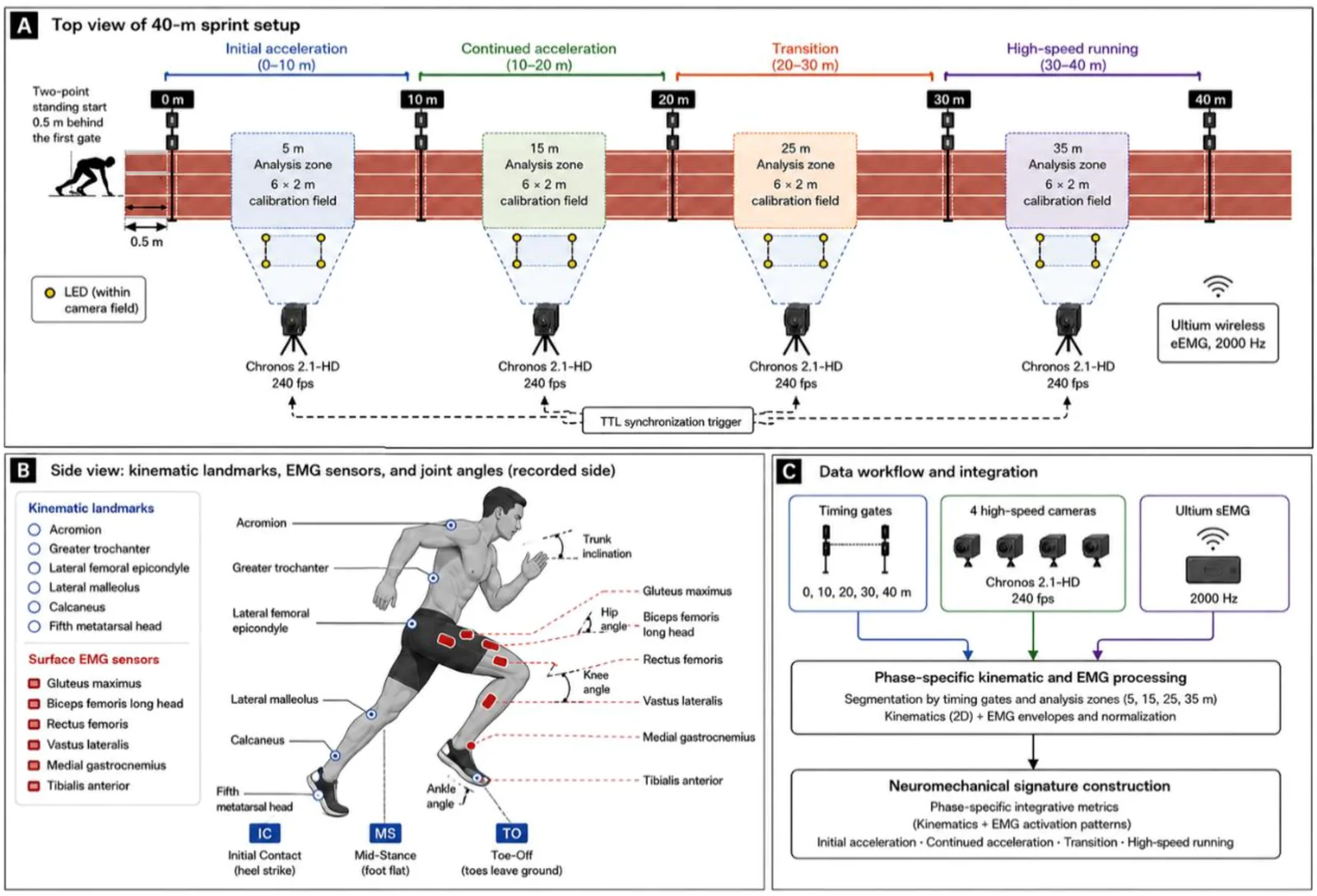
Experimental setup and integrated workflow for phase-specific neuromechanical analysis during a 40-m sprint. **(A)** Top-view schematic of the sprint protocol showing the standing-start position, dual-beam timing gates at 0, 10, 20, 30, and 40 m, four operational sprint phases, calibrated analysis zones centred at 5, 15, 25, and 35 m, high-speed camera placement, wireless sEMG acquisition, and TTL-based synchronization. **(B)** Sagittal-plane anatomical landmarks, surface-electromyography sensor locations, joint-angle definitions, and discrete stride events used for analysis: initial contact (IC), mid-stance (MS), and toe-off (TO). **(C)** Data-integration workflow linking timing, two-dimensional kinematics, and sEMG signals to phase-specific neuromechanical processing and signature construction. sEMG, surface electromyography; TTL, transistor–transistor logic.

### Neuromechanical variables and signal processing

The primary trial selected according to the sprint protocol was processed. Within each sprint phase, the complete stride of the recorded leg whose temporal midpoint was closest to the centre of the calibrated camera field was analyzed. The same prespecified recorded leg was retained across all four sprint phases for each participant, thereby maintaining a consistent within-participant reference for kinematic, sEMG, coactivation, and excitation–movement coupling measures. Restricting extraction to one standardized limb also preserved the participant as the primary sampling unit and avoided introducing additional within-participant limb dependence that would have required explicit bilateral modelling. Because the study was designed to characterize phase-specific neuromechanical performance patterns rather than inter-limb asymmetry, no *post hoc* selection of the limb showing the more favorable sprint mechanics or muscle-excitation profile was permitted. The same initial-contact and toe-off boundaries were applied to the corresponding kinematic and sEMG signals to preserve temporal alignment between movement mechanics and muscle excitation. Kinematic analysis was performed using Dartfish 10 (Dartfish SA, Fribourg, Switzerland). Marker coordinates were digitized frame by frame, exported as numerical time-series data, and filtered in R using a fourth-order zero-lag Butterworth low-pass filter with a 12-Hz cut-off frequency. Initial contact was defined as the first video frame showing visible foot–ground contact, toe-off as the first frame following complete loss of ground contact, and mid-stance as 50% of stance duration.

Ground-contact time was calculated from initial contact to toe-off, whereas flight time was calculated from toe-off to the subsequent contralateral initial contact. Step length was defined as the horizontal distance between consecutive contralateral foot contacts and normalized to leg length. Step frequency was calculated as the inverse of step time. Trunk inclination and hip, knee, and ankle angles were extracted at initial contact, mid-stance, and toe-off within each sprint phase. Peak joint angles and ranges of motion were calculated across the stance phase, while continuous joint-angle waveforms were analyzed separately across stance and swing. For waveform-level analyses, continuous trunk-inclination and hip-, knee-, and ankle-angle trajectories were retained across the complete stance and swing periods rather than being reduced to discrete event values. Trunk inclination was defined relative to the global vertical axis; hip angle was formed by the trunk and thigh segments, knee angle by the thigh and shank segments, and ankle angle by the shank and foot segments. Positive angular directions were defined *a priori* for forward trunk inclination, hip extension, knee extension, and plantar flexion. The operational definitions, units, and normalization procedures for the extracted performance, spatiotemporal, kinematic, electromyographic, and neuromechanical-coupling variables are summarized in **Table 2**.

**Table 2:** Operational definitions of the performance, kinematic, electromyographic, and neuromechanical-coupling variables.

Domain	Variable	Operational definition	Unit or normalization
Performance	Total sprint velocity	40 m divided by total electronic sprint time	m·s^-^¹
Performance	Interval velocity	10 m divided by the corresponding split time	m·s^-^¹
Spatiotemporal	Ground-contact time	Time from initial contact to toe-off	s
Spatiotemporal	Flight time	Time from toe-off to subsequent contralateral initial contact	s
Spatiotemporal	Step length	Horizontal distance between consecutive contralateral contacts	Leg-length normalized
Spatiotemporal	Step frequency	Inverse of step time	Hz
Kinematic	Trunk inclination	Acromion–greater trochanter segment relative to vertical	Degrees
Kinematic	Hip angle	Angle formed by trunk and thigh segments	Degrees
Kinematic	Knee angle	Angle formed by thigh and shank segments	Degrees
Kinematic	Ankle angle	Angle formed by shank and foot segments	Degrees
sEMG amplitude	Mean excitation	Mean processed amplitude within the selected phase	%MVIC
sEMG amplitude	Integrated EMG	Area under the normalized excitation curve	%MVIC·s
sEMG timing	Time to peak excitation	Time from phase onset to peak excitation	ms or % phase
sEMG timing	Activation duration	Duration between excitation onset and offset	ms or % phase
Coordination	Coactivation index	Integrated ratio of lower to higher simultaneous muscle-pair amplitude	%
Coupling	Excitation–event offset	Temporal difference between an sEMG landmark and a kinematic event	ms

Raw sEMG signals were processed using MyoResearch 3 (Noraxon USA Inc., Scottsdale, AZ, USA). Signals were demeaned, band-pass filtered between 20 and 450 Hz using a fourth-order zero-lag Butterworth filter, full-wave rectified, and smoothed using a 50-ms root-mean-square window. Amplitudes were normalized to the highest sTable 500-ms epoch obtained during the corresponding maximal voluntary isometric contraction and expressed as a percentage of that reference. MVIC normalization was selected as the primary amplitude-normalization procedure to provide a participant- and muscle-specific physiological reference and to improve comparability of sEMG amplitude across participants by reducing the influence of non-physiological differences related to electrode placement, skin–electrode characteristics, and absolute signal magnitude. The highest sTable 500-ms epoch was used rather than a single instantaneous peak to reduce the influence of transient signal spikes while retaining a period representative of maximal voluntary activation.

Muscle excitation was quantified during three prespecified temporal windows: the 100 ms preceding initial contact, the complete stance phase, and the subsequent swing phase. Outcomes included mean normalized amplitude, integrated EMG, peak amplitude, activation duration, and time to peak excitation. Activation onset and offset were identified when the processed signal crossed 20% of the within-stride peak and remained above or below that threshold for at least 25 ms. The 20% within-stride peak criterion was prespecified to provide a relative, muscle- and stride-specific threshold that was sufficiently above low-amplitude baseline fluctuations while remaining sensitive to the rapid excitation changes characteristic of sprinting. Using a within-stride relative threshold also reduced dependence of onset and offset detection on between-participant differences in absolute sEMG amplitude. The additional 25-ms persistence criterion was imposed to prevent brief threshold crossings caused by residual noise or transient fluctuations from being classified as physiological activation onset or offset. These detection criteria were applied identically to all six muscles, participants, sprint phases, and analyzed strides, without participant-specific manual adjustment.

In addition to these discrete sEMG outcomes, the complete processed excitation waveforms from the gluteus maximus, biceps femoris long head, rectus femoris, vastus lateralis, medial gastrocnemius, and tibialis anterior were retained for waveform-level statistical analysis.

For Statistical Parametric Mapping, stance and swing were treated as distinct biomechanical periods and were independently time-normalized from 0% to 100% using 101 equally spaced points. Thus, 0% and 100% represented initial contact and toe-off, respectively, for stance, and toe-off and the subsequent initial contact, respectively, for swing. Time normalization was performed separately for every participant, sprint phase, kinematic waveform, and sEMG waveform before group-level statistical analysis. Waveforms from the four sprint phases—initial acceleration, continued acceleration, transition, and high-speed running—were not concatenated; each sprint phase was analyzed independently to preserve phase-specific temporal structure.

The waveform-level dataset therefore comprised trunk, hip, knee, and ankle kinematic trajectories together with the six normalized sEMG trajectories, separately represented over stance and swing within each sprint phase. The same preprocessing and event definitions were applied to both training groups before statistical comparison. Waveform-level SPM analyses were complementary to the discrete-outcome and predictive-model analyses: individual waveform time points were not entered as candidate predictors in the elastic-net neuromechanical signature models and were not used to determine which discrete features entered those models.

Coactivation was calculated for the biceps femoris–rectus femoris, biceps femoris–vastus lateralis, and medial gastrocnemius–tibialis anterior muscle pairs. At each normalized time point, the lower amplitude was divided by the higher amplitude, and the resulting ratio was integrated over the corresponding movement phase and expressed as a percentage. Neuromechanical coupling variables comprised the temporal offsets between excitation onset and initial contact, peak excitation and mid-stance, and excitation offset and toe-off. These event-linked measures, together with the discrete kinematic and sEMG outcomes, constituted the candidate feature set. Because MVIC obtained under isometric conditions may not fully reproduce the excitation characteristics of rapid dynamic sprinting, a second sEMG dataset normalized to the highest sprint-derived amplitude for the corresponding muscle was generated as a prespecified sensitivity analysis. This alternative normalization was used to determine whether the principal sEMG and integrated-model findings were robust to the choice of amplitude reference rather than dependent specifically on MVIC normalization.

### Measurement reliability and quality assurance

Camera calibration, timing-gate alignment, electrode fixation, wireless signal quality, and synchronization accuracy were verified before each testing session. A trial was repeated following a false start, slip, premature deceleration, timing-system failure, synchronization error exceeding one video frame, substantial marker occlusion, sensor displacement, signal saturation, or irrecoverable sEMG artefact. Testing was discontinued if a participant reported pain, dizziness, or other symptoms incompatible with maximal sprinting. All files were coded before processing, and assessors were blinded to group classification and sprint-time outcomes. Within-session reliability was evaluated across the three valid trials for total and split times, principal kinematic outcomes, and primary sEMG measures.

A stratified random subsample of 40 participants, comprising 20 participants from each group, was reprocessed by the same assessor after 7–14 days to determine intra-rater reliability. A second trained assessor independently processed 20 of these datasets to determine inter-rater reliability. Within-session and intra-rater reliability were quantified using ICC(3, 1), whereas inter-rater reliability was assessed using ICC(2, 1). Intraclass correlation coefficients were reported with 95% confidence intervals. Absolute measurement error was quantified using the standard error of measurement, coefficient of variation, and minimal detectable change at the 95% confidence level. Kinematic event selections differing by more than one video frame were reviewed and redigitized. sEMG channels were inspected for excessive baseline noise, signal dropout, clipping, movement artefact, and abnormal spectral content. Variables with an ICC below 0.75 or a coefficient of variation exceeding 20% were excluded from signature development. Individual sEMG channels failing the quality criteria were removed without excluding otherwise valid kinematic data. Participants were excluded from integrated modelling only when the required multimodal feature set could not be reconstructed. All trial exclusions, signal corrections, and redigitization decisions were documented before model development.

### Neuromechanical signature construction

The full candidate feature pool was defined before reliability screening and comprised 332 phase-specific predictors: 16 spatiotemporal features, 64 non-spatiotemporal segmental and joint kinematic features, 168 sEMG amplitude and timing features, 12 coactivation features, and 72 excitation–movement coupling features. Because measurements obtained in different sprint phases were treated as distinct predictors, these counts refer to phase-specific feature instances rather than unique variable names. Reliability screening excluded two sEMG features that did not satisfy the predefined criteria (ICC ≥ 0.75 and coefficient of variation ≤20%), leaving 330 reliability-qualified candidate features. The excluded features were tibialis anterior activation duration during initial acceleration and medial gastrocnemius time to peak excitation during continued acceleration. Candidate features were organized by sprint phase into kinematic, sEMG, and neuromechanical-coupling domains. A complete domain-by-domain accounting of the candidate pool and feature-selection pipeline is provided in **Supplementary Table S4**.

Separate outcome-specific models were developed. The primary performance-related signature was derived using mean 40-m sprint velocity as the outcome. Secondary models were developed for individual interval velocities and sprint-training-status classification. Feature processing proceeded sequentially through reliability screening, separation of spatiotemporal benchmark variables, training-fold standardization, correlation filtering, elastic-net regularization, and stability assessment. Continuous features were standardized as z-scores using parameters estimated from the corresponding model-training data. Within each outer training fold, predictors showing high pairwise correlation ( ∣r∣≥0.90) were reduced by retaining the variable with greater measurement reliability and clearer biomechanical interpretability. Because this procedure was repeated independently within each training fold, the exact number of retained predictors varied across resamples.

To address the mathematical dependence between running velocity and its underlying spatiotemporal components, normalized step length, step frequency, ground-contact time, and flight time were treated separately from the primary neuromechanical candidate feature set for continuous velocity modelling. Step length and step frequency represent direct constituents of running velocity, whereas contact and flight times are closely related to the temporal organization underlying step frequency; their inclusion as ordinary predictors could therefore inflate apparent predictive performance through mathematical or structural overlap with the outcome. The 16 phase-specific spatiotemporal features were therefore separated from the primary performance-related candidate pool, leaving 314 reliability-qualified non-spatiotemporal neuromechanical features. A direct spatiotemporal benchmark was therefore constructed from normalized step length and step frequency, and an extended benchmark additionally included ground-contact time and flight time. The primary neuromechanical models were restricted to segmental and joint kinematics, sEMG features, coactivation, and excitation–movement coupling variables.

For continuous sprint-velocity outcomes, the primary neuromechanical comparisons included a non-spatiotemporal kinematic model, an sEMG-only model, and an integrated neuromechanical model, together with the direct and extended spatiotemporal benchmarks. Augmented models combined the integrated neuromechanical feature set with each spatiotemporal benchmark to determine whether neuromechanical information improved prediction beyond the direct and proximal spatiotemporal components of running velocity.

To quantify the incremental contribution of muscle-excitation and coupling information beyond basic kinematic parameters, an additional nested modelling sequence was evaluated. Before fold-specific correlation filtering, the non-spatiotemporal kinematic, sEMG, kinematics + sEMG, and fully integrated candidate pools contained 64, 166, 230, and 314 features, respectively. After correlation filtering, the median numbers retained across outer training folds were 38 (range 35–41), 71 (65–77), 96 (89–103), and 121 (112–131), respectively. The first model contained only non-spatiotemporal segmental and joint kinematic features. The second model added phase-specific sEMG amplitude and excitation features while excluding coactivation and excitation–movement coupling variables. The third, fully integrated model additionally incorporated coactivation and excitation–movement coupling features. Comparisons between the first and second models quantified the incremental information provided by sEMG beyond kinematics, whereas comparisons between the second and third models quantified the additional contribution of neuromechanical coupling and coordination variables. For sprint-training-status classification, for which mathematical overlap with velocity was not applicable, the full reliability-qualified feature set was retained.

Elastic-net regularization was used to identify parsimonious combinations of correlated predictors. Univariate statistical significance was not used as a feature-selection criterion. Across the internal-validation resamples of the fully integrated performance model, the elastic-net solution contained a median of 22 non-zero coefficients (IQR 18–26). Selection frequency for each feature was calculated as the proportion of internal-validation resamples in which its coefficient was non-zero. A feature was considered signature-stable when it received a non-zero coefficient in at least 70% of internal-validation resamples and demonstrated a consistent coefficient direction. Nine of the 314 non-spatiotemporal candidate features ultimately met these stability criteria and constituted the primary performance-related signature. All feature-processing and selection procedures were embedded within the resampling framework described in statistical analysis. Standardization, correlation filtering, elastic-net selection, and hyperparameter tuning were repeated within the corresponding training data so that information from the outer test folds did not enter feature selection. Following internal validation, a descriptive model was refitted to the complete analytical sample using the selected hyperparameters. A composite signature score was calculated as the weighted sum of the retained standardized features. For the primary performance-related signature, this score was derived only from retained non-spatiotemporal neuromechanical features, thereby preventing direct components of running velocity from dominating the interpretation of the signature. The contribution of each sprint phase was examined to determine whether performance was associated with a common neuromechanical organization or distinct phase-dependent configurations. For descriptive phase-level interpretation, the contribution of each phase was calculated as the sum of the absolute standardized coefficients of stable features originating from that phase divided by the summed absolute standardized coefficients across all stable features, expressed as a percentage. These phase contribution percentages therefore represent relative shares of coefficient magnitude within the stable signature and should not be interpreted as proportions of variance explained, independent phase effects, or phase-specific *R*^2^. Performance estimates were derived exclusively from out-of-fold predictions and not from the refitted descriptive model.

### Statistical analysis

Statistical analyses were performed in R version 4.4.3 (R Foundation for Statistical Computing, Vienna, Austria), with elastic-net models fitted using the glmnet package version 4.1-8. Waveform-level one-dimensional statistical parametric mapping was conducted in Python version 3.12.8 using spm1d version 0.4.53. A master random seed of 20260317 was specified before generation of the repeated cross-validation partitions, with subsequent resampling seeds generated deterministically from this master seed to ensure reproducibility of fold allocation, model tuning, and bootstrap procedures. Continuous variables were summarized as mean ± standard deviation or median and interquartile range, as appropriate. Categorical variables were reported as frequencies and percentages. Baseline group characteristics were described using standardized mean differences. Distributional assumptions were evaluated using residual plots, Q–Q plots, and influence diagnostics. Statistical tests were two-sided, with significance set at α = 0.05, and effect estimates were reported with 95% confidence intervals. Discrete phase-specific kinematic and sEMG outcomes were analyzed using linear mixed-effects models. Sprint-training group, sprint phase, and their interaction were entered as fixed effects, and participant was included as a random intercept. Sex was included as a prespecified covariate.

For models in which a neuromechanical variable was the dependent outcome, interval velocity was centered within each sprint phase and included as an additional covariate. Velocity was not entered as a covariate when it was the dependent variable. Prespecified contrasts were estimated from marginal means and adjusted using the Holm procedure. Results were reported as adjusted mean differences, standardized effect estimates, 95% confidence intervals, and marginal and conditional *R*^2^. Time-normalized kinematic and sEMG waveforms were examined separately within each sprint phase using one-dimensional general linear models. Group was entered as the primary factor, with sex and interval velocity included as covariates. For each kinematic and sEMG waveform, the general linear model was fitted across the 101 normalized time points, producing a one-dimensional statistical parametric field over the 0–100% movement cycle. The prespecified contrast tested the adjusted difference between sprint-trained athletes and physically active non-sprinters. Analyses were conducted independently for the initial-acceleration, continued-acceleration, transition, and high-speed-running phases, with stance and swing treated as separate waveform domains.

Statistical inference for the waveform analyses was performed using random field theory as implemented in spm1d. Residual smoothness was estimated from the model residuals and incorporated into determination of the critical threshold for each one-dimensional statistical field. A two-sided family-wise error rate of $p_{\text{FWE}}<0.05$was applied within each waveform. Contiguous portions of the trajectory exceeding the corresponding random-field-theory threshold were identified as supra-threshold clusters, and statistical significance was determined using cluster-level probabilities, with $p_{\text{FWE}}<0.05$indicating a significant temporal region. The Benjamini–Hochberg procedure was additionally applied across related muscle and joint waveform families to control the false-discovery rate across waveform-level tests. Thus, random-field-theory inference controlled the temporal search across each complete waveform, whereas the Benjamini–Hochberg procedure addressed multiplicity across related waveform families. Significant regions were reported according to normalized temporal location, cluster extent, cluster-level *p*-value, and direction of the adjusted between-group difference. The beginning and end of each supra-threshold region were expressed as percentages of the normalized stance or swing cycle. Waveform-level SPM analyses were conducted as complementary inferential analyses and were not used to select candidate predictors for the elastic-net models. Individual normalized waveform time points therefore did not enter the predictive feature-selection pipeline.

Mean 40-m sprint velocity was designated as the primary multivariable outcome. Interval velocities were treated as secondary continuous outcomes, whereas sprint-training-status classification was treated as a secondary exploratory analysis. Because the number of candidate predictors exceeded the complete multimodal analytical sample size, the regularized models were regarded as exploratory, internally validated model-development analyses rather than externally validated or prospectively validated prediction models. Elastic-net linear regression was used for continuous velocity outcomes, whereas elastic-net logistic regression was used for sprint-training-status classification. Sex was entered as an unpenalized covariate. The prespecified candidate pool comprised 332 phase-specific features: 16 spatiotemporal features, 64 non-spatiotemporal kinematic features, 168 sEMG amplitude and timing features, 12 coactivation features, and 72 excitation–movement coupling features. Reliability screening removed two sEMG features, leaving 330 reliability-qualified features before outcome-specific model construction.

For continuous velocity modelling, five core models were evaluated to distinguish the direct mathematical components of running velocity from additional neuromechanical information. The direct spatiotemporal benchmark comprised normalized step length and step frequency, which are mathematical constituents of running velocity. An extended spatiotemporal benchmark additionally included ground-contact time and flight time to account for closely related temporal organization. For the primary continuous-velocity neuromechanical models, the 16 phase-specific spatiotemporal features were separated from the candidate pool, leaving 314 reliability-qualified non-spatiotemporal features eligible for modelling. The remaining models comprised a non-spatiotemporal kinematic model containing segmental and joint kinematic features, an sEMG model, and an integrated neuromechanical model combining non-spatiotemporal kinematics, sEMG, coactivation, and excitation–movement coupling features.

To evaluate the incremental contribution of sEMG and coupling information beyond basic kinematics, an additional nested intermediate model combined the non-spatiotemporal kinematic features with sEMG amplitude and excitation features but excluded coactivation and excitation–movement coupling variables. Thus, the hierarchical sequence comprised (1) non-spatiotemporal kinematics, (2) kinematics + sEMG, and (3) the fully integrated model including kinematics, sEMG, coactivation, and excitation–movement coupling. Before fold-specific correlation filtering, these three nested models contained 64, 230, and 314 reliability-qualified candidate features, respectively; the sEMG-only model contained 166 features.

Two augmented models were additionally evaluated. The first combined the direct spatiotemporal benchmark with the integrated neuromechanical feature set, whereas the second combined the extended spatiotemporal benchmark with the integrated neuromechanical feature set. These augmented models were used to determine whether the integrated neuromechanical feature set provided incremental concurrent predictive information beyond the mathematical constituents of running velocity and whether such information persisted after additionally accounting for ground-contact and flight times.

Model development and internal evaluation used nested five-fold cross-validation repeated 10 times, yielding 50 outer training–test partitions for each outcome. Outer folds were generated at the participant level and stratified jointly by sprint-training group and sex. Each participant therefore appeared in an outer test fold once during each repetition and generated 10 out-of-fold predictions across the complete repeated procedure. Identical outer-fold assignments were used for all models compared for a given outcome, thereby ensuring paired evaluation on the same test observations. Outer-test observations were not used during preprocessing, correlation filtering, feature selection, hyperparameter tuning, classification-threshold specification, or stability assessment.

All feature-processing steps, including standardization, correlation filtering, feature selection, and hyperparameter tuning, were conducted independently within the corresponding training data. Continuous predictors were standardized within each outer training set using the means and standard deviations estimated from that training set, and these fixed scaling parameters were subsequently applied to the corresponding outer test observations without re-estimation.

Within each outer training set, predictors with an absolute pairwise correlation of ∣r∣≥0.90were reduced according to a deterministic prespecified rule. For each correlated pair, the feature with the higher intraclass correlation coefficient was retained; if reliability coefficients were equivalent, the feature with the lower coefficient of variation was retained, and any remaining exact tie was resolved according to the prespecified ordering of variables in the feature dictionary. No outer-test outcomes, test-fold prediction errors, or *post hoc* biomechanical judgments were used to determine which correlated feature was retained. Because correlation filtering was repeated independently within each training fold, the number of predictors entering elastic-net estimation varied across resamples; for the fully integrated performance model, a median of 121 features (range 112–131) remained after this step.

Within each outer training set, elastic-net hyperparameters were optimized exclusively through the inner cross-validation loop, preventing information from the outer test fold from influencing model development or tuning. The elastic-net mixing parameter *α* was evaluated from 0.10 to 1.00 in increments of 0.02. For each *α*, 100 logarithmically spaced λvalues were evaluated between $\lambda_{\text{max}}$, defined as the smallest penalty yielding all penalized coefficients equal to zero, and $0.001\times \lambda_{\text{max}}$. For continuous outcomes, tuning was based on mean inner-fold RMSE; for binary classification, mean binomial deviance was used. To favor parsimonious solutions in the high-dimensional setting, the final penalty was selected using the one-standard-error rule, whereby the largest λyielding inner-cross-validation error within one standard error of the minimum was retained. The selected *α*– λ combination was then refitted on the complete outer training set and evaluated once on the untouched outer test fold.

Across the fully integrated continuous-outcome models, the median selected *α* was 0.62 (IQR 0.54–0.70). The fitted elastic-net models retained a median of 22 predictors with non-zero coefficients per internal-validation resample (IQR 18–26). Feature-selection stability was evaluated across the 50 independently fitted outer-training models generated by the five outer folds repeated 10 times. Selection frequency was defined as the number of these 50 fitted models in which a candidate received a non-zero coefficient divided by 50. A feature was considered signature-stable when it was selected in at least 70% of the 50 outer-training fits and showed a consistent coefficient direction whenever selected. Nine non-spatiotemporal features satisfied these criteria for the final primary performance-related signature. The complete accounting of candidate features and sequential filtering steps is reported in **Supplementary Table S4**.

Continuous-outcome performance was evaluated primarily using root-mean-square error (RMSE) and additionally using cross-validated *R*^2^ and mean absolute error (MAE). For each participant, the 10 out-of-fold predictions generated across the repeated outer cross-validation procedure were averaged to obtain one participant-level aggregated out-of-fold prediction. Final RMSE, MAE, and cross-validated *R*^2^ were calculated from these aggregated out-of-fold predictions and the corresponding observed outcomes. Cross-validated *R*^2^ was calculated as $1-\sum_{i}{\left(y_{i}-{\hat{y}}_{i}\right)}^{2}/\sum_{i}{\left(y_{i}-\bar{y}\right)}^{2}$.

The primary assessment of incremental neuromechanical value compared the augmented direct-benchmark model with the direct spatiotemporal benchmark, whereas the corresponding comparison using the extended benchmark was treated as a robustness analysis. Within the nested neuromechanical sequence, the kinematics + sEMG model was compared with the non-spatiotemporal kinematic model to quantify the incremental predictive contribution of sEMG, and the fully integrated model was subsequently compared with the kinematics + sEMG model to quantify the additional contribution of coactivation and excitation–movement coupling variables. Changes in cross-validated *R*^2^, RMSE, and MAE were reported for each sequential comparison.

Uncertainty in continuous and classification performance estimates was quantified using 2, 000 participant-level bootstrap resamples of the aggregated observed–out-of-fold-prediction pairs. The full feature-selection and model-development pipeline was not rerun within each bootstrap replicate; instead, the bootstrap quantified sampling uncertainty in the fixed internally validated out-of-fold predictions. For paired model comparisons, identical participant indices were resampled simultaneously for both models so that the within-participant paired structure was retained. Percentile 95% confidence intervals were obtained for *R*^2^, RMSE, MAE, AUC, balanced accuracy, calibration slope, and paired between-model differences.

Evidence of incremental neuromechanical predictive information was based primarily on paired out-of-fold RMSE differences rather than changes in apparent model fit. Incremental value was considered supported when the augmented model produced a lower RMSE than its corresponding benchmark and the 95% confidence interval for the paired RMSE reduction excluded zero. The same criterion was applied to the nested comparisons of kinematics versus kinematics + sEMG and kinematics + sEMG versus the fully integrated model. Changes in cross-validated *R*^2^ and MAE were reported descriptively as complementary measures of the magnitude and consistency of improvement. The non-spatiotemporal integrated neuromechanical model was also compared with the non-spatiotemporal kinematic and sEMG models to evaluate whether multimodal integration improved prediction independently of the direct spatiotemporal constituents of velocity.

Sprint-training-status classification was analyzed as a secondary exploratory objective. Because sprint-training status is not mathematically defined by sprint velocity or its spatiotemporal constituents, classification analyses retained the kinematic-only, sEMG-only, and integrated profiles using all reliability-qualified features. Accordingly, all 330 reliability-qualified features, including the 16 phase-specific spatiotemporal features, remained eligible for the integrated sprint-training-status classification model before fold-specific filtering and regularization.

Classification probabilities were generated exclusively for outer-test participants using models developed within the corresponding training data. A fixed predicted-probability threshold of 0.50 was prespecified for calculation of balanced accuracy, sensitivity, and specificity and remained unchanged across outer folds, repetitions, and compared models. The threshold was not optimized using outer-test observations. Repeated out-of-fold probabilities for each participant were averaged across the 10 repetitions before calculation of the final classification metrics. AUC, Brier score, and calibration slope were calculated from the aggregated continuous predicted probabilities and therefore did not depend on the 0.50 classification threshold. Exploratory paired comparisons of AUC were performed between the integrated and single-modality models using the same participant-level bootstrap framework.

Sensitivity analyses repeated the primary models using the mean of the two fastest trials, sprint-derived sEMG normalization, and exclusion of influential observations identified through standardized residuals and Cook’s distance. The extended spatiotemporal benchmark provided an additional robustness analysis to determine whether the incremental contribution of neuromechanical features persisted when ground-contact and flight times were considered alongside step length and step frequency. The nested kinematic → kinematics + sEMG → fully integrated comparison was also examined across the principal sensitivity analyses to assess whether the incremental contributions of sEMG and coupling variables were robust to alternative analytical conditions. Corrupted biomechanical signals were not imputed. Single-modality analyses included all participants with valid data for the relevant modality, whereas integrated analyses required complete data for the selected multimodal features. Exploratory feature-level analyses were controlled using the Benjamini–Hochberg false-discovery-rate procedure.

Following internal validation, a descriptive final model was refitted to the complete analytical sample using the selected modelling specification for interpretation and reproducible scoring; performance estimates reported in the Results were derived exclusively from out-of-fold predictions and not from this full-sample refit. The final reproducibility materials report the refitted model intercept, all retained coefficients, predictor definitions, centering and scaling parameters, selected hyperparameters, and de-identified outer-fold membership, together with the analysis code and a reproducible scoring specification. These materials permit independent reconstruction of the reported model while preserving participant anonymity.

## Results

### Participant characteristics and data completeness

All 160 participants completed the sprint-testing protocol and contributed valid total and interval timing data. The sprint-trained and physically active non-sprinter groups were balanced by design for sample size and sex distribution. Anthropometric characteristics were broadly comparable between groups, with absolute standardized mean differences ranging from 0.05 to 0.33. As expected from the prespecified classification criteria, sprint-trained athletes demonstrated substantially greater sprint-specific training exposure, including a mean training history exceeding five years and regular maximal-velocity practice. Participant characteristics and modality-specific data availability are presented in **Table 3**. Valid phase-specific kinematic data were available for 158 participants (98.8%), comprising 79 sprint-trained athletes and 79 physically active non-sprinters. Valid synchronized six-muscle sEMG data were available for 154 participants (96.3%), comprising 77 participants per group. The intersection of valid kinematic, sEMG, and synchronization data comprised 152 participants (95.0%; 76 participants per group). This complete synchronized multimodal sample was used for excitation–movement coupling analyses and direct comparisons among the kinematic-only, sEMG-only, and integrated neuromechanical models.

**Table 3:** Participant characteristics and data completeness according to sprint-training status.

Characteristic	Overall (n=160)	Sprint-trained athletes (n=80)	Physically active non-sprinters (n=80)	Standardized mean difference
Age, years	20.5 ± 1.6	20.6 ± 1.5	20.4 ± 1.6	0.13
Women, (n) (%)	80 (50.0)	40 (50.0)	40 (50.0)	0.00
Men, (n) (%)	80 (50.0)	40 (50.0)	40 (50.0)	0.00
Body height, m	1.73 ± 0.08	1.74 ± 0.08	1.72 ± 0.08	0.25
Body mass, kg	67.0 ± 9.6	67.8 ± 9.4	66.1 ± 9.7	0.18
Body mass index, kg·m(^{-2})	22.3 ± 2.2	22.3 ± 2.1	22.2 ± 2.3	0.05
Leg length, m	0.93 ± 0.05	0.93 ± 0.05	0.92 ± 0.05	0.20
Thigh length, m	0.43 ± 0.03	0.43 ± 0.03	0.42 ± 0.03	0.33
Shank length, m	0.42 ± 0.03	0.42 ± 0.03	0.41 ± 0.03	0.33
Right-leg dominant, (n) (%)	142 (88.8)	72 (90.0)	70 (87.5)	0.08
Sprint-training history, years	—	5.4 ± 1.7	—	—
Sprint-training frequency, sessions·week(^{-1})	—	4.7 ± 0.8	—	—
Maximal-velocity exposure, sessions·week(^{-1})	—	1.8 ± 0.6	—	—
Resistance training, sessions·week(^{-1})	—	2.1 ± 0.7	—	—
Plyometric training, sessions·week(^{-1})	—	1.5 ± 0.6	—	—
Recreational moderate-to-vigorous physical activity, min·week(^{-1})	—	—	226 ± 64	—
Valid total and interval timing data, (n) (%)	160 (100)	80 (100)	80 (100)	—
Valid phase-specific kinematic data, (n) (%)	158 (98.8)	79 (98.8)	79 (98.8)	—
Complete six-muscle sEMG data, (n) (%)	154 (96.3)	77 (96.3)	77 (96.3)	—
Complete integrated dataset, (n) (%)	152 (95.0)	76 (95.0)	76 (95.0)	—

Eight participants were excluded from integrated analyses: four because of irrecoverable sEMG artefact, two because of substantial marker occlusion affecting the required kinematic variables, and two because of synchronization failure between the video and sEMG systems. Synchronization failures prevented reliable phase-specific alignment of muscle-excitation signals with the corresponding kinematic events and therefore precluded inclusion in the synchronized sEMG and integrated analyses. Data completeness was balanced between groups, and participants excluded from integrated analyses did not differ materially from those retained in age, sex distribution, anthropometric characteristics, or mean 40-m sprint velocity. No participant withdrew after testing commenced, and no adverse events occurred. Participant-level data availability is illustrated in **Supplementary Figure S1**.

### Measurement reliability and data quality

All participants completed three valid sprint trials. Across the 480 initially scheduled trials, 27 attempts (5.6%) required repetition because of transient marker occlusion ( n=9), sEMG artefact or sensor displacement ( n=8), timing-gate triggering failure ( n=5), synchronization error exceeding one video frame ( n=3), or slipping or premature deceleration ( n=2). All repeated trials were completed successfully after the prescribed recovery period. Accepted trials demonstrated synchronization accuracy within one video frame, with no evidence of signal saturation or systematic temporal drift across the four analysis zones. Sprint timing demonstrated excellent within-session reliability. The ICC for total 40-m sprint time was 0.995, with a coefficient of variation of 0.7%, whereas phase-specific velocity ICCs ranged from 0.987 to 0.994. Spatiotemporal measures also showed consistently high reliability across sprint phases, with ICCs of 0.91–0.96 for ground-contact time, 0.88–0.94 for flight time, 0.90–0.95 for normalized step length, and 0.89–0.94 for step frequency.

Kinematic outcomes demonstrated good-to-excellent reproducibility. Across the 40 datasets reprocessed by the same assessor, intra-rater ICCs ranged from 0.86 to 0.95 for trunk, hip, and knee angles and from 0.82 to 0.90 for ankle angles. Inter-rater agreement in the 20 independently processed datasets ranged from 0.80 to 0.93. Angular standard errors of measurement ranged from 1.3° to 3.1°, corresponding to ${\text{MDC}}_{95}$ values of 3.6°–8.6°. Event identification differed by no more than one video frame in 96.8% of repeated observations; the remaining observations were reviewed and redigitized according to the prespecified quality-control procedure. Reliability of the primary sEMG outcomes was lower than that of timing and kinematic measures but remained acceptable for most phase-specific variables. Within-session ICCs ranged from 0.79 to 0.91 for normalized amplitude and integrated EMG, from 0.76 to 0.88 for activation-timing outcomes, and from 0.79 to 0.90 for coactivation indices. Corresponding coefficients of variation were 8.7%–15.8%, 10.4%–18.9%, and 9.1%–16.6%, respectively. Intra- and inter-rater estimates showed similar ranges, indicating that most measurement variability reflected trial-to-trial biological variation rather than signal-processing inconsistency. Two phase-specific variables did not satisfy the prespecified reliability criteria: tibialis anterior activation duration during initial acceleration (ICC = 0.72; CV = 22.1%) and medial gastrocnemius time to peak excitation during continued acceleration (ICC = 0.73; CV = 20.8%). These variables were excluded before signature development. All remaining candidate features met the minimum criterion of ICC≥0.75 and CV ≤ 20%. Eight individual sEMG channels across six participants were excluded because of irrecoverable artefact; valid data from the remaining muscles and kinematic modality were retained for modality-specific analyses. Reliability and measurement-error estimates are summarized in **Table 4**.

**Table 4:** Reliability and measurement-error estimates for sprint performance, kinematic, and sEMG outcomes.

Outcome domain	Within-session ICC	Intra-rater ICC	Inter-rater ICC	CV, %	SEM	MDC(_{95})
Total 40-m sprint time	0.995	0.998	0.997	0.7	0.03 s	0.08 s
Phase-specific velocity	0.987–0.994	0.991–0.996	0.989–0.995	0.8–1.6	0.05–0.11 m·s(^{-1})	0.14–0.30 m·s(^{-1})
Ground-contact time	0.91–0.96	0.93–0.97	0.90–0.95	2.8–5.1	0.004–0.007 s	0.011–0.019 s
Flight time	0.88–0.94	0.90–0.95	0.87–0.93	3.4–6.2	0.004–0.008 s	0.011–0.022 s
Normalized step length	0.90–0.95	0.92–0.96	0.89–0.94	2.4–4.6	0.02–0.04 leg lengths	0.06–0.11 leg lengths
Step frequency	0.89–0.94	0.91–0.95	0.88–0.93	2.6–4.8	0.09–0.15 Hz	0.25–0.42 Hz
Trunk inclination	0.86–0.93	0.88–0.95	0.84–0.92	4.1–6.8	1.3°–2.0°	3.6°–5.5°
Hip angle	0.84–0.92	0.86–0.94	0.82–0.91	4.6–7.4	1.8°–2.7°	5.0°–7.5°
Knee angle	0.87–0.94	0.89–0.95	0.85–0.93	4.2–6.9	1.7°–2.5°	4.7°–6.9°
Ankle angle	0.82–0.90	0.84–0.92	0.80–0.89	5.8–8.7	2.1°–3.1°	5.8°–8.6°
Normalized sEMG amplitude	0.79–0.90	0.80–0.92	0.77–0.89	9.2–15.8	4.8–8.5% MVIC	13.3–23.6% MVIC
Integrated sEMG	0.80–0.91	0.81–0.92	0.78–0.90	8.7–15.1	4.4–8.0% of mean	12.2–22.2% of mean
Activation-timing outcomes	0.76–0.88	0.77–0.90	0.75–0.87	10.4–18.9	7–15 ms	19–42 ms
Coactivation indices	0.79–0.90	0.80–0.91	0.77–0.89	9.1–16.6	2.7%–5.9%	7.5%–16.4%

### Total and phase-specific sprint performance

Sprint-trained athletes completed the 40-m sprint substantially faster than physically active non-sprinters (5.20 ± 0.31 vs 5.95 ± 0.40 s). After adjustment for sex, the between-group difference in total sprint time was −0.74 s (95% CI −0.85 to −0.63; p<0.001; Hedges’ g=−2.05). Correspondingly, mean 40-m velocity was higher in the sprint-trained group (7.72 ± 0.46 vs 6.75 ± 0.45 m·s^-1^), with an adjusted difference of 0.96 m·s^-1^ (95% CI 0.83 to 1.09; p<0.001; g=2.10). The linear mixed-effects model identified significant effects of sprint-training group ( $F_{1,157}=186.42$, p<0.001), sprint phase ( $F_{3,471}=1642.18$, p<0.001), and group × phase interaction ( $F_{3,471}=31.76$, p<0.001). The fixed effects explained 83% of the variance in interval velocity (marginal $R^{2}=0.83$), while the complete model explained 89% (conditional $R^{2}=0.89$). The interaction remained evident after adjustment for sex, indicating that the between-group difference varied systematically across the sprint.

Sprint-trained athletes achieved higher adjusted interval velocities in every phase. The smallest difference occurred during initial acceleration (adjusted mean difference, 0.48 m·s^-1^; 95% CI 0.38 to 0.58), whereas progressively larger differences were observed during continued acceleration (1.04 m·s^-1^; 95% CI 0.90 to 1.18), transition (1.18 m·s^-1^; 95% CI 1.02 to 1.34), and high-speed running (1.78 m·s^-1^; 95% CI 1.59 to 1.97). All Holm-adjusted comparisons were significant at p<0.001. Within-group contrasts showed that velocity increased across all successive intervals in the sprint-trained group (all Holm-adjusted p<0.001). Physically active non-sprinters also demonstrated marked increases from initial to continued acceleration and from continued acceleration to transition (both p<0.001); however, the increase from transition to high-speed running was comparatively small and did not reach the adjusted significance threshold ( p=0.084). Thus, the principal divergence between groups emerged after 20 m, when sprint-trained athletes continued to increase velocity while non-sprinters approached a performance plateau. Total and phase-specific sprint outcomes are presented in **Table 5**, and the velocity profiles are illustrated in **Figure 2**.

**Table 5:** Total and phase-specific sprint performance according to sprint-training status.

Performance outcome	Sprint-trained athletes (n=80)	Physically active non-sprinters (n=80)	Adjusted mean difference (95% CI)	Adjusted (p)-value	Standardized effect
Total 40-m time, s	5.20 ± 0.31	5.95 ± 0.40	−0.74 (−0.85 to −0.63)	<0.001	−2.05
Mean 40-m velocity, m·s(^{-1})	7.72 ± 0.46	6.75 ± 0.45	0.96 (0.83 to 1.09)	<0.001	2.10
0–10-m time, s	1.89 ± 0.11	2.08 ± 0.13	−0.19 (−0.23 to −0.15)	<0.001	−1.57
Initial-acceleration velocity, m·s(^{-1})	5.30 ± 0.32	4.82 ± 0.31	0.48 (0.38 to 0.58)	<0.001	1.52
10–20-m time, s	1.23 ± 0.07	1.41 ± 0.09	−0.18 (−0.20 to −0.15)	<0.001	−2.20
Continued-acceleration velocity, m·s(^{-1})	8.15 ± 0.44	7.11 ± 0.46	1.04 (0.90 to 1.18)	<0.001	2.30
20–30-m time, s	1.08 ± 0.06	1.25 ± 0.08	−0.16 (−0.19 to −0.14)	<0.001	−2.26
Transition velocity, m·s(^{-1})	9.23 ± 0.48	8.05 ± 0.49	1.18 (1.02 to 1.34)	<0.001	2.42
30–40-m time, s	1.00 ± 0.05	1.23 ± 0.09	−0.22 (−0.25 to −0.20)	<0.001	−3.01
High-speed-running velocity, m·s(^{-1})	9.98 ± 0.53	8.20 ± 0.56	1.78 (1.59 to 1.97)	<0.001	3.24

**Figure 2:**
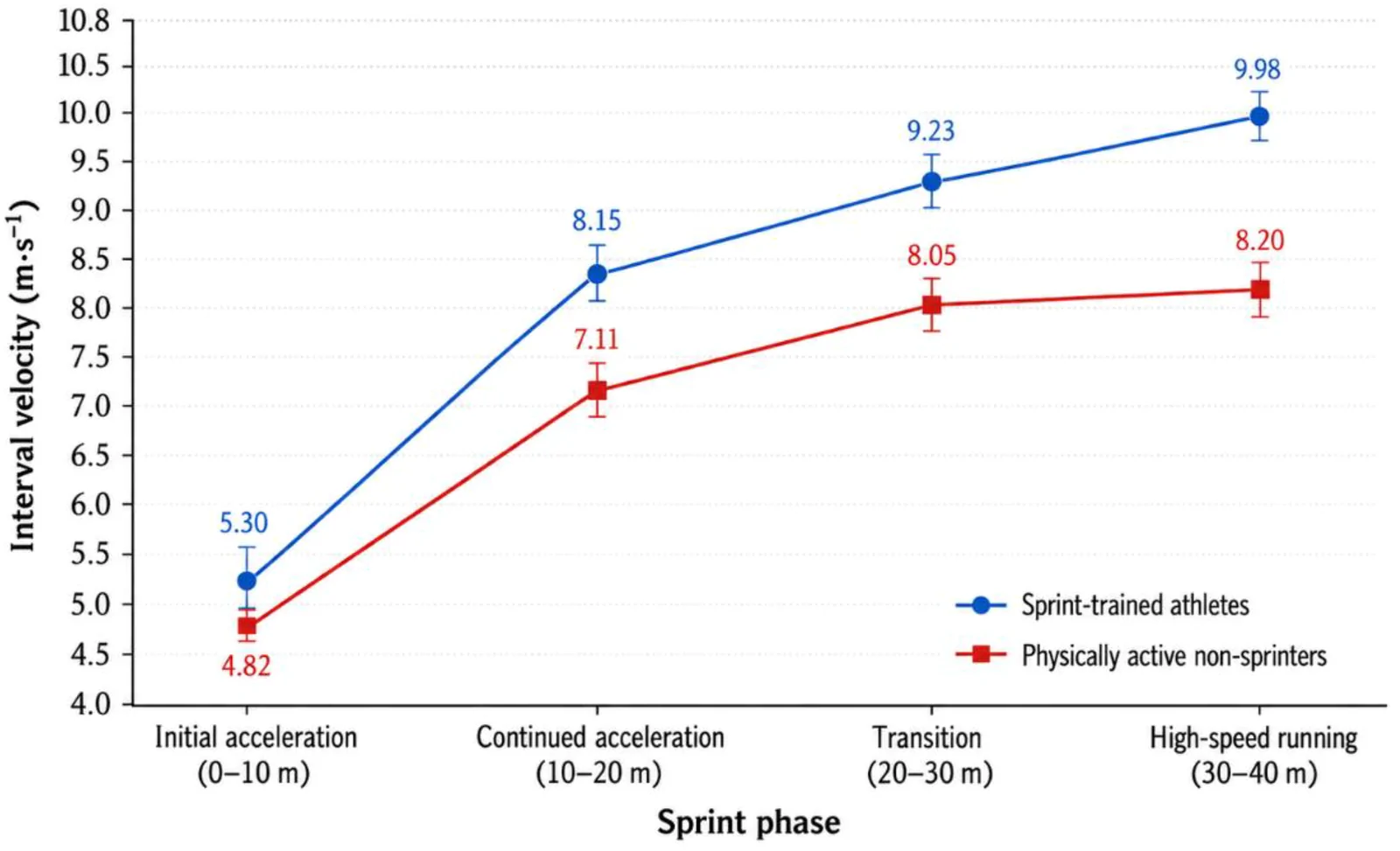
Phase-specific sprint velocity profiles in sprint-trained athletes and physically active non-sprinters.

### Phase-specific discrete kinematic outcomes

Valid phase-specific kinematic data were available for 158 participants (79 per group). After adjustment for sex and phase-centered interval velocity, significant group × phase interactions were identified for normalized step length ( $F_{3,468}=14.82$, p<0.001), step frequency ( $F_{3,468}=6.91$, p<0.001), ground-contact time ( $F_{3,468}=12.64$, p<0.001), and flight time ( $F_{3,468}=8.37$, p<0.001). Significant phase effects were observed for all spatiotemporal outcomes (all p<0.001), indicating systematic reorganization of step mechanics as the sprint progressed. Sprint-trained athletes demonstrated longer normalized step lengths in every phase, with the adjusted between-group difference increasing from 0.08 leg lengths during initial acceleration to 0.26 leg lengths during high-speed running. Step frequency did not differ during initial acceleration but was progressively higher in the sprint-trained group from continued acceleration onward. Ground-contact time was consistently shorter in sprint-trained athletes, with the largest adjusted difference occurring during transition and high-speed running. Flight time was correspondingly longer, although the between-group separation was modest during initial acceleration and became more pronounced after 20 m.

Discrete joint and segment kinematics also exhibited phase-dependent group differences. Significant group × phase interactions were identified for trunk inclination at initial contact ( $F_{3,468}=17.29$, p<0.001), knee angle at initial contact ( $F_{3,468}=9.44$, p<0.001), hip extension at toe-off ( $F_{3,468}=7.81$, p<0.001), and ankle plantar flexion at initial contact ( $F_{3,468}=6.58$, p<0.001). The corresponding marginal *R*^2^ values ranged from 0.42 to 0.71, while conditional *R*^2^ values ranged from 0.61 to 0.84. During initial and continued acceleration, sprint-trained athletes contacted the ground with greater forward trunk inclination than non-sprinters. This pattern reversed during transition and high-speed running, where sprint-trained athletes adopted a more upright trunk orientation. Across all phases, the sprint-trained group demonstrated a more extended knee at initial contact, with the adjusted difference increasing from 4.8° during initial acceleration to 10.7° during high-speed running. Hip extension at toe-off and ankle plantar flexion at initial contact were also greater in sprint-trained athletes, particularly after 20 m. These differences remained after adjustment for interval velocity, indicating that the observed mechanical organization was not explained solely by the higher running speeds attained by sprint-trained athletes. No consistent adjusted group differences were observed for hip angle at mid-stance, ankle range of motion, or knee angle at mid-stance after Holm correction. Selected phase-specific outcomes are presented in **Table 6**; the complete discrete kinematic results are provided in **Supplementary Table S2**.

**Table 6:** Adjusted phase-specific discrete kinematic outcomes according to sprint-training status.

Outcome	Sprint phase	Sprint-trained athletes (n=79)	Physically active non-sprinters (n=79)	Adjusted difference (95% CI)	Holm-adjusted (p)
Normalized step length, leg lengths	Initial acceleration	1.38 ± 0.03	1.30 ± 0.03	0.08 (0.03 to 0.13)	0.002
	Continued acceleration	1.83 ± 0.03	1.69 ± 0.03	0.14 (0.08 to 0.20)	<0.001
	Transition	2.03 ± 0.04	1.82 ± 0.04	0.21 (0.14 to 0.28)	<0.001
	High-speed running	2.13 ± 0.04	1.87 ± 0.04	0.26 (0.19 to 0.33)	<0.001
Step frequency, Hz	Initial acceleration	3.73 ± 0.05	3.68 ± 0.05	0.05 (−0.04 to 0.14)	0.286
	Continued acceleration	4.22 ± 0.06	4.04 ± 0.06	0.18 (0.07 to 0.29)	0.001
	Transition	4.45 ± 0.06	4.19 ± 0.06	0.26 (0.14 to 0.38)	<0.001
	High-speed running	4.53 ± 0.06	4.21 ± 0.06	0.32 (0.20 to 0.44)	<0.001
Ground-contact time, s	Initial acceleration	0.171 ± 0.003	0.184 ± 0.003	−0.013 (−0.019 to −0.007)	<0.001
	Continued acceleration	0.136 ± 0.003	0.153 ± 0.003	−0.017 (−0.023 to −0.011)	<0.001
	Transition	0.116 ± 0.004	0.139 ± 0.004	−0.023 (−0.030 to −0.016)	<0.001
	High-speed running	0.107 ± 0.004	0.131 ± 0.004	−0.024 (−0.031 to −0.017)	<0.001
Flight time, s	Initial acceleration	0.071 ± 0.003	0.064 ± 0.003	0.007 (0.001 to 0.013)	0.018
	Continued acceleration	0.091 ± 0.004	0.079 ± 0.004	0.012 (0.005 to 0.019)	0.001
	Transition	0.108 ± 0.004	0.089 ± 0.004	0.019 (0.011 to 0.027)	<0.001
	High-speed running	0.116 ± 0.004	0.095 ± 0.004	0.021 (0.013 to 0.029)	<0.001
Trunk inclination at initial contact, °	Initial acceleration	43.2 ± 1.1	39.0 ± 1.1	4.2 (1.6 to 6.8)	0.002
	Continued acceleration	31.1 ± 1.2	27.3 ± 1.2	3.8 (1.1 to 6.5)	0.006
	Transition	20.1 ± 1.3	23.2 ± 1.3	−3.1 (−5.9 to −0.3)	0.029
	High-speed running	14.2 ± 1.3	19.6 ± 1.3	−5.4 (−8.2 to −2.6)	<0.001
Knee angle at initial contact, °	Initial acceleration	150.1 ± 1.3	145.3 ± 1.3	4.8 (1.9 to 7.7)	0.001
	Continued acceleration	156.4 ± 1.5	149.7 ± 1.5	6.7 (3.4 to 10.0)	<0.001
	Transition	162.0 ± 1.7	153.1 ± 1.7	8.9 (5.2 to 12.6)	<0.001
	High-speed running	166.3 ± 1.8	155.6 ± 1.8	10.7 (6.9 to 14.5)	<0.001
Hip extension at toe-off, °	Initial acceleration	9.3 ± 0.9	7.2 ± 0.9	2.1 (0.2 to 4.0)	0.031
	Continued acceleration	13.2 ± 1.0	9.2 ± 1.0	4.0 (1.8 to 6.2)	<0.001
	Transition	17.1 ± 1.1	11.0 ± 1.1	6.1 (3.6 to 8.6)	<0.001
	High-speed running	19.0 ± 1.2	12.0 ± 1.2	7.0 (4.4 to 9.6)	<0.001
Ankle plantar flexion at initial contact, °	Initial acceleration	3.2 ± 1.1	0.2 ± 1.1	3.0 (0.5 to 5.5)	0.019
	Continued acceleration	6.4 ± 1.2	2.1 ± 1.2	4.3 (1.6 to 7.0)	0.002
	Transition	9.1 ± 1.3	3.7 ± 1.3	5.4 (2.5 to 8.3)	<0.001
	High-speed running	12.0 ± 1.4	5.2 ± 1.4	6.8 (3.8 to 9.8)	<0.001

### Phase-specific sEMG and neuromechanical-coupling outcomes

Complete six-muscle sEMG data were available for 154 participants, comprising 77 sprint-trained athletes and 77 physically active non-sprinters. These data were used for phase-specific excitation-amplitude, activation-timing, and muscle-coactivation analyses. Synchronized kinematic–sEMG data required for excitation–movement coupling analyses were available for 152 participants, comprising 76 participants per group. Tibialis anterior activation duration during initial acceleration and medial gastrocnemius time to peak excitation during continued acceleration were excluded because they did not meet the prespecified reliability criteria; their removal did not reduce the participant-level analytical samples. After adjustment for sex and phase-centered interval velocity, significant group × phase interactions were observed for gluteus maximus mean stance amplitude ( $F_{3,456}=8.62$, p<0.001), vastus lateralis mean stance amplitude ( $F_{3,456}=7.05$, p<0.001), biceps femoris preactivation ( $F_{3,456}=12.94$, p<0.001), and medial gastrocnemius mean stance amplitude ( $F_{3,456}=14.31$, p<0.001). Gluteus maximus and vastus lateralis excitation was greater in sprint-trained athletes during initial acceleration, whereas the between-group differences diminished in later phases. In contrast, biceps femoris preactivation and medial gastrocnemius stance excitation diverged progressively across the sprint, with the largest adjusted differences occurring during transition and high-speed running. Rectus femoris and tibialis anterior mean amplitudes did not show consistent between-group differences after Holm adjustment.

Muscle-coactivation patterns were also phase dependent. Significant group × phase interactions were identified for biceps femoris–rectus femoris ( $F_{3,456}=9.72$, p<0.001), biceps femoris–vastus lateralis ( $F_{3,456}=8.86$, p<0.001), and medial gastrocnemius–tibialis anterior coactivation ( $F_{3,456}=8.41$, p<0.001). Coactivation was comparable between groups during initial acceleration but became progressively lower in sprint-trained athletes from continued acceleration onward. The largest adjusted differences were observed during high-speed running, particularly for the biceps femoris–rectus femoris and medial gastrocnemius–tibialis anterior pairs. Neuromechanical-coupling analyses demonstrated significant group × phase interactions for biceps femoris excitation onset relative to initial contact ( $F_{3,450}=11.57$, p<0.001), medial gastrocnemius excitation onset relative to initial contact ( $F_{3,450}=10.18$, p<0.001), biceps femoris peak-excitation timing relative to mid-stance ( $F_{3,450}=7.46$, p<0.001), and medial gastrocnemius excitation offset relative to toe-off ( $F_{3,450}=5.93$, p=0.001). Sprint-trained athletes demonstrated earlier biceps femoris and medial gastrocnemius excitation before initial contact, particularly after 20 m. Peak biceps femoris excitation also occurred closer to mid-stance, whereas medial gastrocnemius excitation persisted closer to toe-off during high-speed running. Marginal *R*^2^ values ranged from 0.38 to 0.67 for the sEMG and coactivation models and from 0.41 to 0.63 for the coupling models. Corresponding conditional *R*^2^ values ranged from 0.57 to 0.81 and from 0.59 to 0.79, respectively. Overall, the adjusted results identified a phase-specific reorganization of muscle excitation rather than a uniform increase in activation across the sprint. Early acceleration was characterized primarily by greater hip- and knee-extensor excitation, whereas transition and high-speed running were distinguished by greater hamstring preactivation, greater plantar-flexor excitation, earlier pre-contact excitation, and reduced antagonist coactivation. Selected outcomes are presented in **Table 7**.

**Table 7:** Selected adjusted phase-specific sEMG and neuromechanical-coupling outcomes according to sprint-training status.

A. sEMG amplitude and coactivation outcomes n=154: 77 sprint-trained athletes and 77 physically active non-sprinters					
Outcome	Initial acceleration	Continued acceleration	Transition	High-speed running	Group × phase (p)
Gluteus maximus mean stance amplitude, %MVIC	94.1 ± 3.0/82.0 ± 3.0; Δ 12.1 (5.5 to 18.7), (p<0.001)	79.8 ± 2.8/73.6 ± 2.8; Δ 6.2 (0.1 to 12.3), (p=0.046)	65.9 ± 2.7/63.8 ± 2.7; Δ 2.1 (−3.8 to 8.0), (p=0.481)	59.2 ± 2.6/60.1 ± 2.6; Δ −0.9 (−6.6 to 4.8), (p=0.758)	<0.001
Vastus lateralis mean stance amplitude, %MVIC	101.2 ± 3.2/89.0 ± 3.2; Δ 12.2 (5.2 to 19.2), (p<0.001)	87.1 ± 3.0/81.7 ± 3.0; Δ 5.4 (−1.2 to 12.0), (p=0.108)	74.6 ± 2.8/73.7 ± 2.8; Δ 0.9 (−5.2 to 7.0), (p=0.771)	67.0 ± 2.7/68.1 ± 2.7; Δ −1.1 (−7.0 to 4.8), (p=0.714)	<0.001
Biceps femoris preactivation, %MVIC	32.1 ± 1.8/30.0 ± 1.8; Δ 2.1 (−1.8 to 6.0), (p=0.291)	42.3 ± 2.0/36.0 ± 2.0; Δ 6.3 (1.9 to 10.7), (p=0.005)	53.0 ± 2.2/42.1 ± 2.2; Δ 10.9 (6.1 to 15.7), (p<0.001)	61.2 ± 2.4/46.0 ± 2.4; Δ 15.2 (10.0 to 20.4), (p<0.001)	<0.001
Medial gastrocnemius mean stance amplitude, %MVIC	61.3 ± 2.5/58.0 ± 2.5; Δ 3.3 (−2.2 to 8.8), (p=0.238)	76.0 ± 2.7/69.1 ± 2.7; Δ 6.9 (1.0 to 12.8), (p=0.022)	91.2 ± 3.0/77.0 ± 3.0; Δ 14.2 (7.7 to 20.7), (p<0.001)	98.1 ± 3.2/80.2 ± 3.2; Δ 17.9 (10.9 to 24.9), (p<0.001)	<0.001
Biceps femoris–rectus femoris coactivation, %	40.1 ± 1.4/41.0 ± 1.4; Δ −0.9 (−4.0 to 2.2), (p=0.568)	35.2 ± 1.5/39.1 ± 1.5; Δ −3.9 (−7.2 to −0.6), (p=0.021)	30.0 ± 1.6/38.0 ± 1.6; Δ −8.0 (−11.5 to −4.5), (p<0.001)	26.1 ± 1.7/37.0 ± 1.7; Δ −10.9 (−14.6 to −7.2), (p<0.001)	<0.001
Medial gastrocnemius–tibialis anterior coactivation, %	35.0 ± 1.3/36.1 ± 1.3; Δ −1.1 (−4.0 to 1.8), (p=0.454)	31.0 ± 1.4/35.0 ± 1.4; Δ −4.0 (−7.1 to −0.9), (p=0.012)	26.1 ± 1.5/33.0 ± 1.5; Δ −6.9 (−10.2 to −3.6), (p<0.001)	22.0 ± 1.5/31.2 ± 1.5; Δ −9.2 (−12.5 to −5.9), (p<0.001)	<0.001

B. Excitation–movement coupling outcomes n=152: 76 sprint-trained athletes and 76 physically active non-sprinters					
Biceps femoris onset relative to initial contact, ms	−43 ± 3/−40 ± 3; Δ −3 (−9 to 3), (p=0.327)	−55 ± 3/−47 ± 3; Δ −8 (−14 to −2), (p=0.009)	−67 ± 3/−52 ± 3; Δ −15 (−22 to −8), (p<0.001)	−76 ± 4/−55 ± 4; Δ −21 (−29 to −13), (p<0.001)	<0.001
Medial gastrocnemius onset relative to initial contact, ms	−28 ± 3/−27 ± 3; Δ −1 (−7 to 5), (p=0.742)	−35 ± 3/−32 ± 3; Δ −3 (−9 to 3), (p=0.318)	−47 ± 3/−38 ± 3; Δ −9 (−16 to −2), (p=0.011)	−56 ± 4/−41 ± 4; Δ −15 (−23 to −7), (p<0.001)	<0.001
Biceps femoris peak relative to mid-stance, ms	18 ± 3/21 ± 3; Δ −3 (−9 to 3), (p=0.341)	12 ± 3/18 ± 3; Δ −6 (−12 to 0), (p=0.052)	6 ± 3/15 ± 3; Δ −9 (−16 to −2), (p=0.012)	2 ± 3/13 ± 3; Δ −11 (−18 to −4), (p=0.002)	<0.001
Medial gastrocnemius offset relative to toe-off, ms	−18 ± 3/−20 ± 3; Δ 2 (−4 to 8), (p=0.521)	−12 ± 3/−16 ± 3; Δ 4 (−2 to 10), (p=0.183)	−7 ± 3/−14 ± 3; Δ 7 (0 to 14), (p=0.049)	−2 ± 3/−12 ± 3; Δ 10 (3 to 17), (p=0.005)	0.001

### Waveform-level kinematic and sEMG differences

#### Kinematic waveforms

Time-normalized kinematic waveforms were available for 158 participants, comprising 79 sprint-trained athletes and 79 physically active non-sprinters. One-dimensional statistical parametric mapping identified temporally localized group differences that varied across sprint phases after adjustment for sex and phase-specific interval velocity. For each significant region, temporal boundaries indicate the beginning and end of the supra-threshold cluster on the normalized 0–100% stance or swing cycle, and cluster-level probabilities were derived using random-field-theory inference. All reported clusters survived family-wise error correction within each waveform and false-discovery-rate correction across related joint-waveform families.

During initial acceleration, sprint-trained athletes demonstrated greater forward trunk inclination during the first 34% of stance (0–34%; $SPM{\left\{t\right\}}_{max}=4.62$, $p_{\text{cluster}}=0.002$, $q_{\text{FDR}}=0.008$), greater knee extension during early stance (0–21%; $SPM{\left\{t\right\}}_{max}=4.18$, $p_{\text{cluster}}=0.006$, $q_{\text{FDR}}=0.014$), and greater hip extension during late stance (62–100%; $SPM{\left\{t\right\}}_{max}=4.44$, $p_{\text{cluster}}=0.003$, $q_{\text{FDR}}=0.010$). These clusters occupied 34%, 21%, and 38% of the normalized stance cycle, respectively. A similar but shorter trunk-inclination cluster was observed during continued acceleration (0–24%; $SPM{\left\{t\right\}}_{max}=3.87$, $p_{\text{cluster}}=0.012$, $q_{\text{FDR}}=0.022$), accompanied by greater knee extension during the first 18% of stance (0–18%; $SPM{\left\{t\right\}}_{max}=3.64$, $p_{\text{cluster}}=0.019$, $q_{\text{FDR}}=0.031$) and greater hip extension from 58% of stance to toe-off (58–100%; $SPM{\left\{t\right\}}_{max}=4.09$, $p_{\text{cluster}}=0.007$, $q_{\text{FDR}}=0.016$).

The trunk waveform diverged in the opposite direction during transition and high-speed running. Sprint-trained athletes exhibited a more upright trunk during the first 31% of stance in transition (0–31%; $SPM{\left\{t\right\}}_{min}=-4.31$, $p_{\text{cluster}}=0.004$, $q_{\text{FDR}}=0.011$) and during the first 44% of stance in high-speed running (0–44%; $SPM{\left\{t\right\}}_{min}=-4.78$, $p_{\text{cluster}}=0.001$, $q_{\text{FDR}}=0.006$). The negative SPM statistics reflect lower forward-trunk-inclination values in the sprint-trained group, consistent with a more upright posture. In both phases, they also showed greater knee extension during initial contact and early stance, with significant clusters spanning 0–24% during transition ( $SPM{\left\{t\right\}}_{max}=4.56$, $p_{\text{cluster}}=0.003$, $q_{\text{FDR}}=0.010$) and 0–29% during high-speed running ( $SPM{\left\{t\right\}}_{max}=5.01$, $p_{\text{cluster}}<0.001$, $q_{\text{FDR}}=0.004$). Greater hip extension was observed during late stance in transition (61–100%; $SPM{\left\{t\right\}}_{max}=4.27$, $p_{\text{cluster}}=0.005$, $q_{\text{FDR}}=0.013$) and high-speed running (57–100%; $SPM{\left\{t\right\}}_{max}=4.65$, $p_{\text{cluster}}=0.002$, $q_{\text{FDR}}=0.008$).

During high-speed running, the ankle waveform contained two distinct supra-threshold clusters. Sprint-trained athletes exhibited greater plantar flexion during early stance (0–17%; $SPM{\left\{t\right\}}_{max}=3.92$, $p_{\text{cluster}}=0.011$, $q_{\text{FDR}}=0.023$) and again during terminal stance (74–100%; $SPM{\left\{t\right\}}_{max}=4.21$, $p_{\text{cluster}}=0.006$, $q_{\text{FDR}}=0.016$). Swing-phase differences were concentrated after 20 m. Sprint-trained athletes demonstrated greater knee flexion during mid-swing in transition (34–59%; $SPM{\left\{t\right\}}_{min}=-4.11$, $p_{\text{cluster}}=0.008$, $q_{\text{FDR}}=0.017$) and high-speed running (31–63%; $SPM{\left\{t\right\}}_{min}=-4.73$, $p_{\text{cluster}}=0.002$, $q_{\text{FDR}}=0.008$), followed by more rapid knee extension during terminal swing. The terminal-swing extension clusters extended from 82–100% during transition ( $SPM{\left\{t\right\}}_{max}=3.76$, $p_{\text{cluster}}=0.015$, $q_{\text{FDR}}=0.027$) and from 78–100% during high-speed running ( $SPM{\left\{t\right\}}_{max}=4.22$, $p_{\text{cluster}}=0.006$, $q_{\text{FDR}}=0.015$). No corrected group differences were identified for hip-angle waveforms during early swing or for ankle waveforms during initial acceleration. No additional kinematic supra-threshold clusters remained significant after the prespecified within-waveform random-field-theory inference and false-discovery-rate correction across related waveform tests. Collectively, the SPM findings demonstrate that between-group kinematic differences were temporally localized rather than uniformly distributed across the movement cycle, with the principal stance-phase clusters concentrated around initial contact and late propulsion and the principal swing-phase clusters occurring during mid- and terminal swing. Significant kinematic clusters are summarized in **Table 8** and illustrated in **Figure 3**.

**Table 8:** Significant waveform-level kinematic and sEMG differences between sprint-trained athletes and physically active non-sprinters.

Modality	Sprint phase	Waveform	Significant region	Cluster extent	Direction in sprint-trained athletes	SPM statistic	Cluster-level $p_{\text{FWE}}$	FDR-adjusted q
Kinematic	Initial acceleration	Trunk inclination, stance	0–34%	34%	Greater forward inclination	$t_{\text{max}}=4.62$	0.002	0.008
Kinematic	Initial acceleration	Knee angle, stance	0–21%	21%	Greater knee extension	$t_{\text{max}}=4.18$	0.006	0.014
Kinematic	Initial acceleration	Hip angle, stance	62–100%	38%	Greater hip extension	$t_{\text{max}}=4.44$	0.003	0.010
Kinematic	Continued acceleration	Trunk inclination, stance	0–24%	24%	Greater forward inclination	$t_{\text{max}}=3.87$.	0.012	0.022
Kinematic	Continued acceleration	Knee angle, stance	0–18%	18%	Greater knee extension	$t_{\text{max}}=3.64$	0.019	0.031
Kinematic	Continued acceleration	Hip angle, stance	58–100%	42%	Greater hip extension	$t_{\text{max}}=4.09$	0.007	0.016
Kinematic	Transition	Trunk inclination, stance	0–31%	31%	More upright trunk	$t_{\text{min}}=-4.31$	0.004	0.011
Kinematic	Transition	Knee angle, stance	0–24%	24%	Greater knee extension	$t_{\text{max}}=4.56$	0.003	0.010
Kinematic	Transition	Hip angle, stance	61–100%	39%	Greater hip extension	$t_{\text{max}}=4.27$	0.005	0.013
Kinematic	Transition	Knee angle, swing	34–59%	25%	Greater mid-swing knee flexion	$t_{\text{min}}=-4.11$	0.008	0.017
Kinematic	Transition	Knee angle, swing	82–100%	18%	Greater terminal-swing knee extension	$t_{\text{max}}=3.76$	0.015	0.027
Kinematic	High-speed running	Trunk inclination, stance	0–44%	44%	More upright trunk	$t_{\text{min}}=-4.78$	0.001	0.006
Kinematic	High-speed running	Knee angle, stance	0–29%	29%	Greater knee extension	$t_{\text{max}}=5.01$	<0.001	0.004
Kinematic	High-speed running	Hip angle, stance	57–100%	43%	Greater hip extension	$t_{\text{max}}=4.65$	0.002	0.008
Kinematic	High-speed running	Ankle angle, stance	0–17%	17%	Greater plantar flexion	$t_{\text{max}}=3.92$	0.011	0.023
Kinematic	High-speed running	Ankle angle, stance	74–100%	26%	Greater plantar flexion	$t_{\text{max}}=4.21$	0.006	0.016
Kinematic	High-speed running	Knee angle, swing	31–63%	32%	Greater mid-swing knee flexion	$t_{\text{min}}=-4.73$	0.002	0.008
Kinematic	High-speed running	Knee angle, swing	78–100%	22%	Greater terminal-swing knee extension	$t_{\text{max}}=4.22$	0.006	0.015
sEMG	Initial acceleration	Gluteus maximus, stance	7–55%	48%	Greater excitation	$t_{\text{max}}=4.76$	0.001	0.006
sEMG	Initial acceleration	Vastus lateralis, stance	0–43%	43%	Greater excitation	$t_{\text{max}}=4.39$	0.003	0.010
sEMG	Continued acceleration	Biceps femoris, swing	81–100%	19%	Greater excitation	$t_{\text{max}}=3.84$	0.013	0.026
sEMG	Continued acceleration	Medial gastrocnemius, stance	46–100%	54%	Greater excitation	$t_{\text{max}}=4.12$	0.007	0.017
sEMG	Transition	Biceps femoris, swing	72–100%	28%	Greater excitation	$t_{\text{max}}=4.51$	0.003	0.010
sEMG	Transition	Biceps femoris, stance	0–18%	18%	Greater excitation	$t_{\text{max}}=3.97$	0.010	0.021
sEMG	Transition	Medial gastrocnemius, stance	21–100%	79%	Greater excitation	$t_{\text{max}}=4.68$	0.002	0.008
sEMG	High-speed running	Biceps femoris, swing	63–100%	37%	Greater excitation	$t_{\text{max}}=5.18$	<0.001	0.003
sEMG	High-speed running	Biceps femoris, stance	0–24%	24%	Greater excitation	$t_{\text{max}}=4.72$	0.002	0.007
sEMG	High-speed running	Medial gastrocnemius, stance	9–100%	91%	Greater excitation	$t_{\text{max}}=5.06$	<0.001	0.004

**Figure 3:**
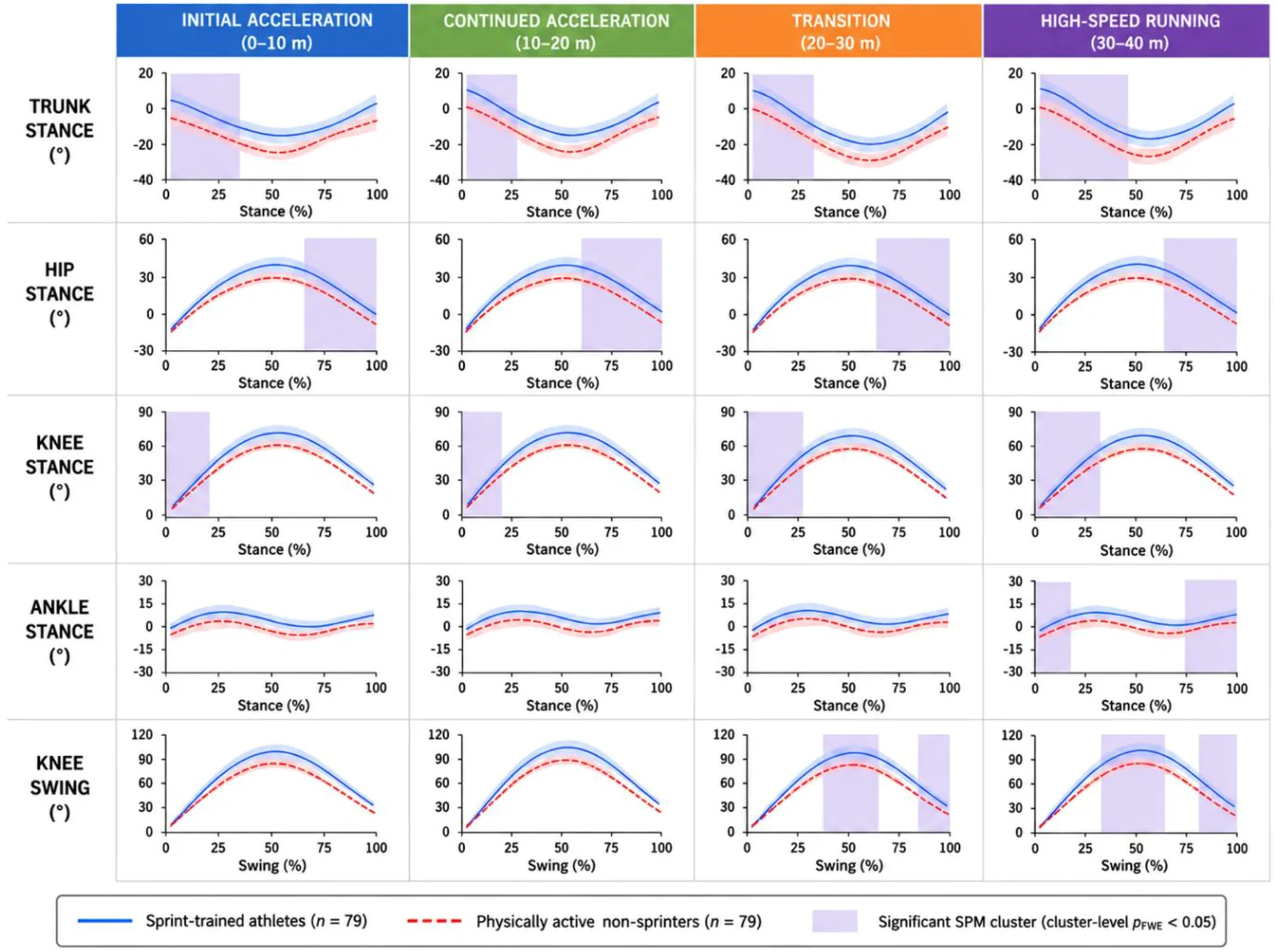
Phase-specific sagittal-plane kinematic waveforms across the 40-m sprint. Mean time-normalized waveforms (95% confidence bands) are shown for sprint-trained athletes ( n=79) and physically active non-sprinters ( n=79) across four sprint phases. Purple shaded regions indicate significant one-dimensional SPM clusters after adjustment for sex and phase-centered interval velocity, with cluster-level $p_{\text{FWE}}<0.05$and FDR-adjusted q<0.05. Exact cluster boundaries and statistics are reported in **Table 8**.

#### sEMG waveforms

Time-normalized sEMG waveforms were available for 154 participants, comprising 77 participants per group. Significant differences were phase- and muscle-specific rather than reflecting a generalized elevation of excitation in sprint-trained athletes. All reported clusters remained significant after random-field-theory family-wise-error control within individual waveforms and Benjamini–Hochberg false-discovery-rate correction across related muscle-waveform tests.

During initial acceleration, sprint-trained athletes demonstrated greater gluteus maximus excitation from 7% to 55% of stance ( $SPM{\left\{t\right\}}_{max}=4.76$, $p_{\text{cluster}}=0.001$, $q_{\text{FDR}}=0.006$) and greater vastus lateralis excitation from initial contact to 43% of stance (0–43%; $SPM{\left\{t\right\}}_{max}=4.39$, $p_{\text{cluster}}=0.003$, $q_{\text{FDR}}=0.010$). These clusters occupied 48% and 43% of the normalized stance cycle, respectively. No corrected biceps femoris or medial gastrocnemius clusters were detected during initial acceleration.

During continued acceleration, biceps femoris excitation was greater in sprint-trained athletes during the final 19% of swing (81–100%; $SPM{\left\{t\right\}}_{max}=3.84$, $p_{\text{cluster}}=0.013$, $q_{\text{FDR}}=0.026$), while medial gastrocnemius excitation was greater from 46% of stance to toe-off (46–100%; $SPM{\left\{t\right\}}_{max}=4.12$, $p_{\text{cluster}}=0.007$, $q_{\text{FDR}}=0.017$). The magnitude and temporal extent of these differences increased during transition. Sprint-trained athletes exhibited greater biceps femoris excitation during terminal swing (72–100%; $SPM{\left\{t\right\}}_{max}=4.51$, $p_{\text{cluster}}=0.003$, $q_{\text{FDR}}=0.010$) and early stance (0–18%; $SPM{\left\{t\right\}}_{max}=3.97$, $p_{\text{cluster}}=0.010$, $q_{\text{FDR}}=0.021$), together with greater medial gastrocnemius excitation across 21–100% of stance ( $SPM{\left\{t\right\}}_{max}=4.68$, $p_{\text{cluster}}=0.002$, $q_{\text{FDR}}=0.008$).

The largest waveform differences occurred during high-speed running. Biceps femoris excitation was greater in sprint-trained athletes during the final 37% of swing (63–100%; $SPM{\left\{t\right\}}_{max}=5.18$, $p_{\text{cluster}}<0.001$, $q_{\text{FDR}}=0.003$) and during the first 24% of stance (0–24%; $SPM{\left\{t\right\}}_{max}=4.72$, $p_{\text{cluster}}=0.002$, $q_{\text{FDR}}=0.007$). Medial gastrocnemius excitation was greater from 9% of stance to toe-off (9–100%; $SPM{\left\{t\right\}}_{max}=5.06$, $p_{\text{cluster}}<0.001$, $q_{\text{FDR}}=0.004$). These clusters occupied 37%, 24%, and 91% of their respective normalized movement periods, indicating increasingly persistent hamstring and plantar-flexor excitation as sprint velocity approached its highest phase.

Gluteus maximus and vastus lateralis waveforms did not differ after correction during transition or high-speed running, and no corrected rectus femoris or tibialis anterior clusters were identified in any sprint phase. No additional supra-threshold sEMG regions remained significant after the prespecified within-waveform random-field-theory inference and false-discovery-rate correction across related muscle-waveform tests.

Collectively, the waveform-level sEMG results demonstrated a phase-dependent redistribution of neuromuscular excitation. Proximal hip- and knee-extensor differences predominated during initial acceleration, whereas biceps femoris preactivation and early-stance excitation, together with progressively sustained medial gastrocnemius excitation, distinguished transition and high-speed running. This temporal localization was consistent with the discrete sEMG outcomes and indicates that group differences reflected specific excitation windows rather than uniformly elevated muscle activity throughout the stride. Significant sEMG clusters are summarized in **Table 8** and illustrated in **Figure 4**.

**Figure 4:**
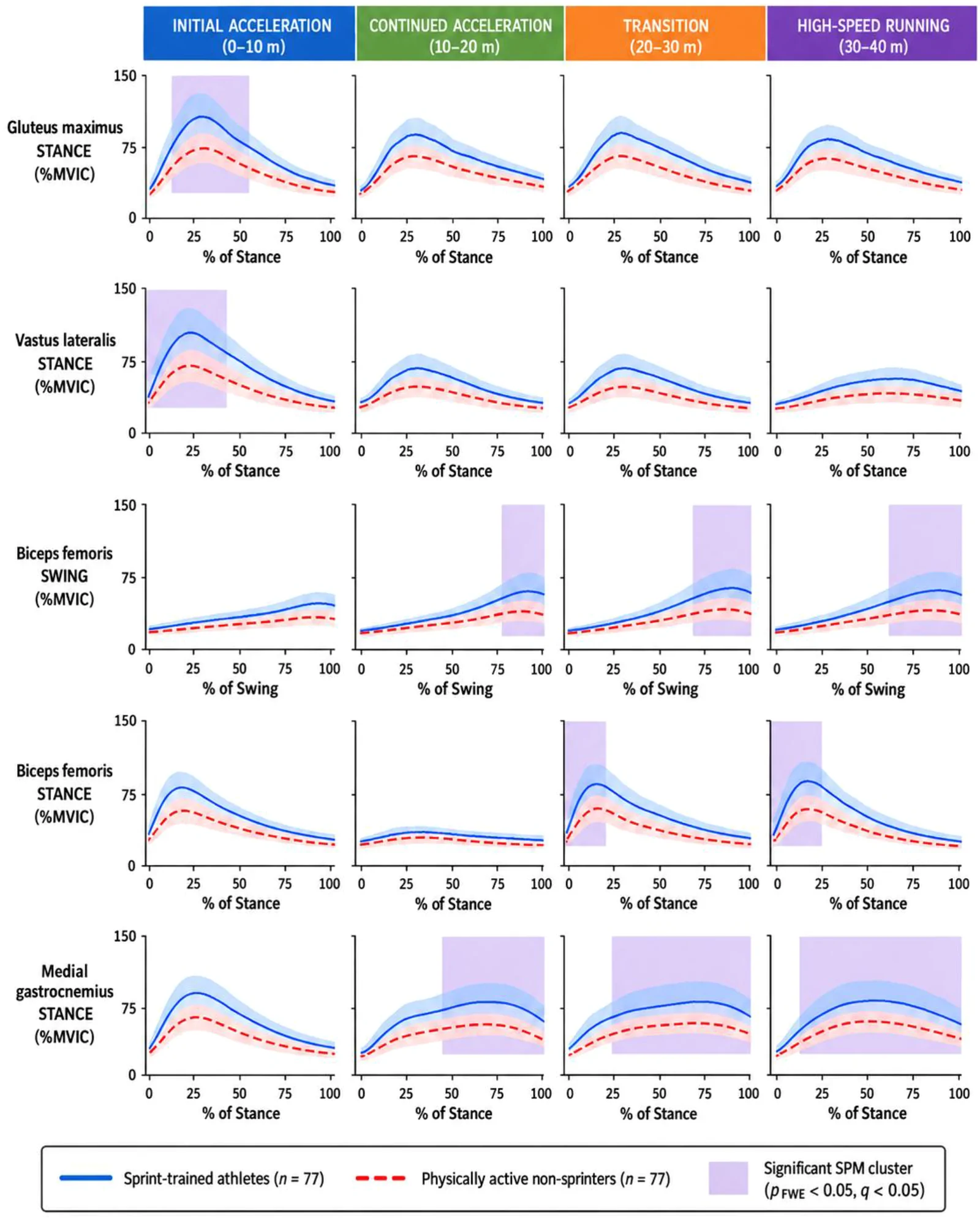
Phase-specific surface-electromyography waveforms across the 40-m sprint. Time-normalized sEMG waveforms are shown for sprint-trained athletes ( n=77) and physically active non-sprinters ( n=77) across four sprint phases. Amplitudes are expressed as %MVIC, with solid and dashed lines representing group means and shaded bands representing 95% confidence intervals. Purple regions indicate significant one-dimensional SPM clusters after adjustment for sex and phase-centered interval velocity ( $p_{\text{FWE}}<0.05$, FDR-adjusted q<0.05). MVIC, maximal voluntary isometric contraction; sEMG, surface electromyography.

### Performance-related neuromechanical signature

The performance-related signature analysis was restricted to the 152 participants with complete synchronized kinematic and sEMG data, comprising 76 sprint-trained athletes and 76 physically active non-sprinters. Identical outer cross-validation folds were used across the direct spatiotemporal benchmark, extended spatiotemporal benchmark, non-spatiotemporal kinematic, sEMG, integrated neuromechanical, and augmented models, ensuring that differences in predictive performance reflected feature content rather than variation in the analytical sample or resampling structure. All continuous-outcome model comparisons were therefore conducted on the same complete-case sample using paired out-of-fold predictions.

### Internally validated model performance

The revised modelling framework showed that the direct spatiotemporal components of running velocity accounted for a substantial proportion of predictive performance but did not fully explain between-participant variation in mean 40-m sprint velocity. The direct spatiotemporal benchmark achieved a cross-validated *R*^2^ of 0.72 (95% CI 0.66–0.77), an RMSE of 0.36 m·s^-1^ (95% CI 0.33–0.40), and an MAE of 0.28 m·s^-1^ (95% CI 0.25–0.31). Adding ground-contact and flight times modestly improved performance, with the extended spatiotemporal benchmark yielding a cross-validated *R*^2^ of 0.76 (95% CI 0.71–0.80), an RMSE of 0.33 m·s^-1^ (95% CI 0.30–0.37), and an MAE of 0.26 m·s^-1^ (95% CI 0.23–0.29).

When the direct and proximal spatiotemporal variables were excluded, the non-spatiotemporal kinematic model explained 60% of the out-of-fold variance in mean 40-m velocity ( $R^{2}=0.60$, 95% CI 0.52–0.67; RMSE = 0.43 m·s^-1^; MAE = 0.34 m·s^-1)^. Adding sEMG amplitude and excitation features increased cross-validated *R*^2^ to 0.69 (95% CI 0.62–0.75), reduced RMSE to 0.38 m·s^-1^ (95% CI 0.35–0.42), and reduced MAE to 0.30 m·s^-1^ (95% CI 0.27–0.33). The paired RMSE reduction of 0.05 m·s^-1^ relative to the kinematic model (95% CI 0.02–0.08) demonstrated incremental predictive information from sEMG beyond segmental and joint kinematics.

Adding coactivation and excitation–movement coupling variables produced a further improvement, with the fully integrated neuromechanical model achieving a cross-validated *R*^2^ of 0.74 (95% CI 0.68–0.79), an RMSE of 0.34 m·s^-1^ (95% CI 0.31–0.38), and an MAE of 0.26 m·s^-1^ (95% CI 0.23–0.29). Relative to the kinematics + sEMG model, this represented an additional RMSE reduction of 0.04 m·s^-1^ (95% CI 0.01–0.07). Thus, the sequential increase in cross-validated *R*^2^ from 0.60 to 0.69 and then to 0.74, together with the corresponding reductions in prediction error, indicated that sEMG and subsequently coupling/coordination variables contributed incremental information beyond basic kinematic parameters. The sEMG-only model explained 55% of the out-of-fold variance ( $R^{2}=0.55$, 95% CI 0.47–0.62; RMSE = 0.45 m·s^-1)^.

The strongest predictive performance was obtained when neuromechanical information was added to the direct spatiotemporal benchmark. The augmented direct-benchmark model achieved a cross-validated *R*^2^ of 0.86 (95% CI 0.82–0.89), an RMSE of 0.25 m·s^-1^ (95% CI 0.23–0.28), and an MAE of 0.20 m·s^-1^ (95% CI 0.18–0.23). This represented an RMSE reduction of 0.11 m·s^-1^ relative to the direct spatiotemporal benchmark (95% CI 0.07–0.15). Because the confidence interval excluded zero, neuromechanical features provided predictive information beyond that attributable to step length and step frequency alone.

The augmented extended-benchmark model produced a similar result, yielding a cross-validated *R*^2^ of 0.87 (95% CI 0.84–0.90), an RMSE of 0.24 m·s^-1^ (95% CI 0.22–0.27), and an MAE of 0.19 (95% CI 0.17–0.22). Relative to the extended spatiotemporal benchmark, RMSE was reduced by 0.09 m·s^-1^ (95% CI 0.06–0.13), indicating that the incremental predictive contribution of neuromechanical information persisted after additional adjustment for ground-contact and flight times. Across the complete nested comparison, the fully integrated model reduced RMSE by 0.09 m·s^-1^ relative to the non-spatiotemporal kinematic model (95% CI 0.05–0.13), of which the sequential comparisons indicated reductions of 0.05 m·s^-1^ after addition of sEMG and a further 0.04 m·s^-1^ after addition of coupling and coordination variables. Prediction errors were similar across training groups and sexes, with no evidence that model performance was driven by one subgroup. Across the outer resampling iterations, the median elastic-net mixing parameter for the integrated neuromechanical model was 0.62 (interquartile range, 0.54–0.70), indicating that the optimal model combined coefficient shrinkage with sparse feature selection. Model performance is summarized in **Table 9**.

**Table 9:** Internally validated performance of the models predicting mean 40-m sprint velocity.

A. Spatiotemporal benchmark and augmented models				
Model	Cross-validated *R*^2^ (95% CI)	RMSE, m·s^-1^ (95% CI)	MAE, m·s^-1^ (95% CI)	Paired RMSE reduction (95% CI)
Direct spatiotemporal benchmark	0.72 (0.66–0.77)	0.36 (0.33–0.40)	0.28 (0.25–0.31)	—
Extended spatiotemporal benchmark	0.76 (0.71–0.80)	0.33 (0.30–0.37)	0.26 (0.23–0.29)	—
Augmented direct-benchmark	0.86 (0.82–0.89)	0.25 (0.23–0.28)	0.20 (0.18–0.23)	0.11 (0.07–0.15) vs direct
Augmented extended-benchmark	0.87 (0.84–0.90)	0.24 (0.22–0.27)	0.19 (0.17–0.22)	0.09 (0.06–0.13) vs extended

B. Incremental contribution of sEMG and coupling variables beyond non-spatiotemporal kinematics				
Non-spatiotemporal kinematic	0.60 (0.52–0.67)	0.43 (0.40–0.47)	0.34 (0.31–0.37)	—
Kinematics + sEMG	0.69 (0.62–0.75)	0.38 (0.35–0.42)	0.30 (0.27–0.33)	0.05 (0.02–0.08) vs kinematic
Fully integrated neuromechanical	0.74 (0.68–0.79)	0.34 (0.31–0.38)	0.26 (0.23–0.29)	0.04 (0.01–0.07) vs kinematics + sEMG
sEMG only	0.55 (0.47–0.62)	0.45 (0.42–0.50)	0.35 (0.32–0.39)	—

### Stable features of the performance signature

The prespecified candidate pool comprised 332 phase-specific features, of which 330 satisfied the reliability criteria after exclusion of two sEMG features. Following separation of the 16 phase-specific spatiotemporal variables for benchmark modelling, 314 reliability-qualified non-spatiotemporal features remained eligible for development of the primary performance-related signature. Fold-specific correlation filtering reduced this pool to a median of 121 predictors (range 112–131), and the elastic-net solutions contained a median of 22 predictors with non-zero coefficients per internal-validation resample (IQR 18–26). From this candidate pool, nine features met the final stability criterion of non-zero selection in at least 70% of internal-validation resamples together with a consistent coefficient direction (**Table 10**). The retained neuromechanical signature comprised three non-spatiotemporal kinematic features, three sEMG features, and three excitation–movement coupling or coordination features. Direct and proximal spatiotemporal variables were not eligible for this primary signature because normalized step length, step frequency, ground-contact time, and flight time were evaluated separately within the benchmark models. A complete domain-specific accounting of the candidate pool and sequential feature-selection pipeline is provided in **Supplementary Table S4**.

**Table 10:** Stable features retained in the integrated performance-related neuromechanical signature.

Sprint phase	Feature	Domain	Selection frequency, %	Standardized coefficient	Association with faster sprint velocity
Initial acceleration	Gluteus maximus mean stance amplitude	sEMG	77	0.11	Greater hip-extensor excitation
Continued acceleration	Hip extension at toe-off	Kinematic	82	0.13	Greater terminal hip extension
Transition	Biceps femoris preactivation	sEMG	91	0.20	Greater pre-contact excitation
Transition	Medial gastrocnemius mean stance amplitude	sEMG	85	0.16	Greater plantar-flexor excitation
High-speed running	Knee angle at initial contact	Kinematic	84	0.15	Greater knee extension
High-speed running	Trunk inclination at initial contact	Kinematic	81	−0.13	More upright trunk
High-speed running	Biceps femoris onset relative to initial contact	Coupling	93	−0.18	Earlier pre-contact onset
High-speed running	Biceps femoris–rectus femoris coactivation	Coordination	89	−0.16	Lower antagonist coactivation
High-speed running	Medial gastrocnemius offset relative to toe-off	Coupling	75	0.10	Excitation maintained closer to toe-off

The largest positive coefficient was observed for biceps femoris preactivation during transition, followed by medial gastrocnemius stance excitation during transition and knee extension at initial contact during high-speed running. Earlier biceps femoris excitation before initial contact, lower biceps femoris–rectus femoris coactivation, greater terminal hip extension, and a more upright trunk orientation were also retained as stable contributors to faster mean 40-m sprint velocity. The inclusion of features from all four sprint phases indicated that the signature was not reducible to a single maximal-speed characteristic. Seven of the nine stable neuromechanical features originated from the transition and high-speed-running phases, indicating that later-phase neuromechanical organization provided the largest share of the non-spatiotemporal performance signature. Importantly, the nine-feature signature was obtained only after reliability screening, fold-specific correlation reduction, and repeated elastic-net stability assessment, rather than through univariate significance testing or selection from the complete dataset. Importantly, these features remained stable after the mathematical constituents of running velocity had been separated from the neuromechanical candidate feature set.

### Phase-specific contributions to the integrated signature

Models restricted to non-spatiotemporal neuromechanical features from a single sprint phase demonstrated progressively stronger prediction from initial acceleration to high-speed running. The initial-acceleration model explained 31% of the out-of-fold variance in mean 40-m velocity, compared with 40% for continued acceleration, 58% for transition, and 68% for high-speed running. However, the all-phase integrated neuromechanical model remained superior to the high-speed-running-only model, increasing cross-validated *R*^2^ from 0.68 to 0.74 and reducing RMSE from 0.38 to 0.34 m·s^-1^. The paired RMSE difference was 0.04 m·s^-1^ (95% CI 0.02–0.06), indicating that information distributed across sprint phases improved prediction beyond high-speed-running features alone.

Based on the summed absolute standardized coefficients of the nine stable neuromechanical features, initial acceleration accounted for approximately 8% of the total coefficient magnitude, continued acceleration 10%, transition 27%, and high-speed running 55%. These values represent relative shares of the summed absolute standardized coefficient magnitude and should not be interpreted as independent proportions of explained variance. Thus, later phases provided the greatest predictive information, while features from initial and continued acceleration retained additional information within the all-phase model. Importantly, these phase-specific patterns were obtained after normalized step length, step frequency, ground-contact time, and flight time had been separated from the primary neuromechanical feature set and evaluated within the benchmark analyses. The internally validated performance of the phase-restricted and all-phase integrated models is presented in **Table 11**. The stable non-spatiotemporal neuromechanical features, standardized coefficients, selection frequencies, and relative phase shares are illustrated in **Figure 5**.

**Table 11:** Internally validated performance of phase-restricted and all-phase integrated models for predicting mean 40-m sprint velocity.

Feature set	Cross-validated *R*^2^	RMSE, m·s^-1^
Initial-acceleration neuromechanical features only	0.31	0.56
Continued-acceleration neuromechanical features only	0.40	0.52
Transition neuromechanical features only	0.58	0.43
High-speed-running neuromechanical features only	0.68	0.38
All-phase integrated neuromechanical features	0.74	0.34

**Figure 5:**
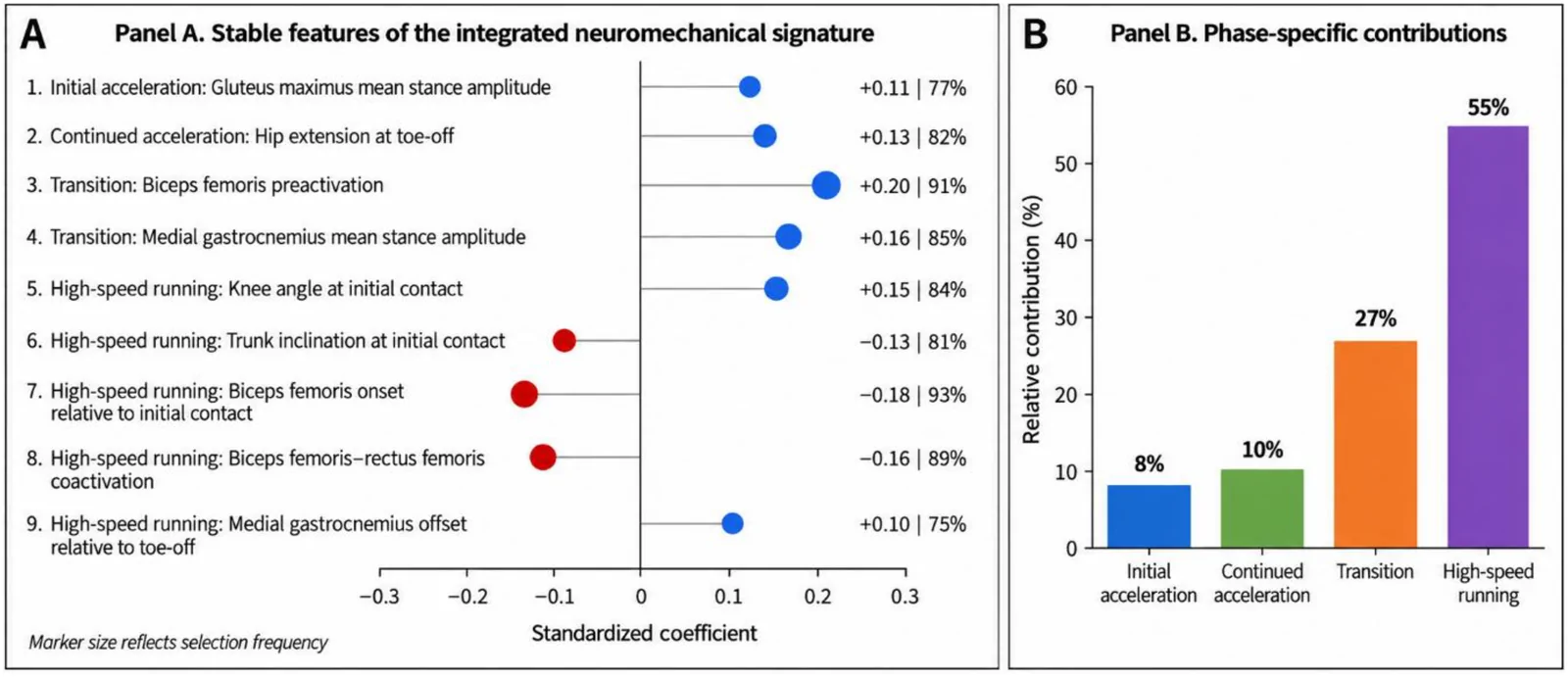
Stable neuromechanical features and phase-specific contributions to the integrated performance signature. **(A)** shows standardized coefficients and selection frequencies of features retained in ≥70% of internal-validation resamples. **(B)** shows the relative share of summed absolute standardized coefficients across sprint phases. Estimates were derived from the complete synchronized multimodal sample ( n=152).

### Sprint-training-status classification

Sprint-training-status classification was evaluated in the complete synchronized multimodal sample of 152 participants, comprising 76 sprint-trained athletes and 76 physically active non-sprinters. Identical outer cross-validation folds were applied to the kinematic-only, sEMG-only, and integrated models, and sex was retained as an unpenalized covariate. Because sprint-training status is not mathematically defined by running velocity or its spatiotemporal constituents, all reliability-qualified features, including step length, step frequency, ground-contact time, and flight time, remained eligible for the classification models. The integrated neuromechanical model demonstrated the highest internally validated discrimination (**Table 12**), with an area under the receiver-operating-characteristic curve of 0.94 (95% CI 0.91–0.97) and balanced accuracy of 0.89 (95% CI 0.85–0.93). The kinematic-only model achieved an AUC of 0.88, whereas the sEMG-only model achieved an AUC of 0.84. Paired bootstrap comparisons confirmed that the integrated model improved AUC by 0.06 relative to the kinematic-only model (95% CI 0.02–0.10) and by 0.10 relative to the sEMG-only model (95% CI 0.05–0.15), satisfying the prespecified superiority criterion.

**Table 12:** Internally validated classification performance for sprint-training status.

Model	AUC (95% CI)	Balanced accuracy (95% CI)	Sensitivity	Specificity	Brier score	Calibration slope
Kinematic only	0.88 (0.83–0.92)	0.82 (0.77–0.87)	0.84	0.80	0.145	0.91
sEMG only	0.84 (0.78–0.89)	0.78 (0.72–0.83)	0.76	0.80	0.168	0.86
Integrated neuromechanical	**0.94 (0.91–0.97)**	**0.89 (0.85–0.93)**	**0.90**	**0.88**	**0.093**	**0.98**

Bold values indicate the best-performing model for each classification metric.

The integrated model correctly identified 90% of sprint-trained athletes and 88% of physically active non-sprinters. Its lower Brier score and calibration slope close to unity indicated more accurate and better-calibrated probability estimates than either single-modality model. Classification performance was similar in men and women, with no evidence of systematic subgroup misclassification. Stable discriminative features were concentrated in the transition and high-speed-running phases. Greater normalized step length, shorter ground-contact time, a more extended knee at initial contact, greater biceps femoris preactivation, earlier biceps femoris excitation onset, greater medial gastrocnemius stance excitation, and lower antagonist coactivation were consistently associated with sprint-trained status. Initial-acceleration gluteus maximus excitation and continued-acceleration hip extension at toe-off also retained independent discriminative value. Seven of the nine stable classification features overlapped with the non-spatiotemporal performance-related signature, whereas normalized step length and ground-contact time were specific to the classification model. This distinction indicates that the characteristics discriminating sprint-training status were largely, but not completely, the same neuromechanical characteristics associated with faster 40-m sprint performance after direct spatiotemporal constituents had been separated from the primary velocity model.

### Sensitivity and robustness analyses

The principal findings were robust across the prespecified alternative analytical conditions. Repeating the performance-related modelling using the mean of the two fastest trials produced results comparable to those obtained from the fastest trial alone. Among the 148 participants whose two fastest trials differed by no more than 2%, the direct spatiotemporal benchmark achieved a cross-validated *R*^2^ of 0.70 and an RMSE of 0.37 m·s^-1^, whereas the augmented direct-benchmark model achieved an *R*^2^ of 0.84 and an RMSE of 0.26 m·s^-1^. The paired RMSE reduction was 0.11 m·s^-1^ (95% CI 0.07–0.15). The extended benchmark and its corresponding augmented model showed a similar pattern ( $R^{2}=0.74$ vs 0.85; RMSE = 0.34 vs 0.25 m·s^-1)^, with a paired RMSE reduction of 0.09 m·s^-1^ (95% CI 0.05–0.13). The nested neuromechanical comparison was also preserved: the non-spatiotemporal kinematic model achieved an *R*^2^ of 0.58 and an RMSE of 0.44 m·s^-1^, addition of sEMG increased *R*^2^ to 0.67 and reduced RMSE to 0.39 m·s^-1^, and subsequent addition of coactivation and excitation–movement coupling increased *R*^2^ to 0.72 and reduced RMSE to 0.36 m·s^-1^. Paired RMSE reductions were 0.05 m·s^-1^ (95% CI 0.02–0.08) for adding sEMG and a further 0.03 m·s^-1^ (95% CI 0.01–0.06) for adding coupling and coordination variables. These findings were consistent with those obtained from the fastest-trial analysis.

Dynamic normalization of sEMG amplitude to the highest sprint-derived value did not materially alter model ranking or predictive performance. Under this normalization approach, the non-spatiotemporal integrated neuromechanical model achieved a cross-validated *R*^2^ of 0.73 and an RMSE of 0.35 m·s^-1^, whereas the augmented direct- and extended-benchmark models achieved *R*^2^ values of 0.85 and 0.86 and RMSE values of 0.26 and 0.25 m·s^-1^, respectively. Within the nested comparison, the kinematic model achieved an *R*^2^ of 0.60 and an RMSE of 0.43 m·s^-1^, the kinematics + sEMG model achieved an *R*^2^ of 0.68 and an RMSE of 0.39 m·s^-1^, and the fully integrated model achieved an *R*^2^ of 0.73 and an RMSE of 0.35 m·s^-1^. The corresponding paired RMSE reductions of 0.04 m·s^-1^ for addition of sEMG and 0.04 m·s^-1^ for addition of coupling variables remained supported by confidence intervals excluding zero. The corresponding classification model achieved an AUC of 0.93 and balanced accuracy of 0.88. Phase-specific mixed-effects estimates for the principal sEMG outcomes changed by less than 8%, and all previously significant group × phase interactions retained the same direction.

Influence diagnostics identified five participants with comparatively large standardized residuals or Cook’s distance values. Excluding these observations reduced the integrated analytical sample to 147 participants but produced only minor changes in model performance. The augmented direct-benchmark model achieved a cross-validated *R*^2^ of 0.87 and an RMSE of 0.24 m·s^-1^, whereas the augmented extended-benchmark model achieved an *R*^2^ of 0.88 and an RMSE of 0.23 m·s^-1^. In both cases, the incremental improvement over the corresponding spatiotemporal benchmark remained statistically supported by paired bootstrap confidence intervals excluding zero. The sequential neuromechanical pattern was likewise maintained after exclusion of influential observations, with *R*^2^ increasing from 0.62 for the kinematic model to 0.70 after addition of sEMG and to 0.75 after addition of coupling and coordination variables; corresponding RMSE values decreased from 0.42 to 0.37 and 0.33 m·s^-1^, respectively. The classification AUC remained 0.94, and the calibration slope remained close to unity. These results indicated that the primary findings were not driven by a small number of extreme observations.

Analyses using all valid modality-specific data yielded similar results to the complete-case comparison. In the full kinematic sample ( n=158), the non-spatiotemporal kinematic model achieved a cross-validated *R*^2^ of 0.61 and an RMSE of 0.42 m·s^-1^. In the full sEMG sample ( n=154), the sEMG-only model achieved a cross-validated *R*^2^ of 0.56 and an RMSE of 0.44 m·s^-1^. Restricting these models to the common complete-case sample of 152 participants resulted in only trivial differences ( $R^{2}=0.60$, RMSE = 0.43 m·s^-1^ for non-spatiotemporal kinematics; $R^{2}=0.55$, RMSE = 0.45 m·s^-1^ for sEMG), confirming that the multimodal findings were not attributable to unequal analytical samples.

Feature stability was also preserved. Eight of the nine primary non-spatiotemporal neuromechanical features remained above the 70% selection threshold across all sensitivity analyses, and all nine retained the same coefficient direction. Transition-phase biceps femoris preactivation, medial gastrocnemius stance excitation, high-speed-running knee angle at initial contact, biceps femoris excitation onset, and biceps femoris–rectus femoris coactivation were selected in more than 80% of resamples under all analytical conditions. High-speed-running medial gastrocnemius offset relative to toe-off showed the greatest variability, falling below the 70% stability threshold in one sensitivity analysis while retaining the same coefficient direction. Overall, the model ranking, incremental predictive value beyond the direct and extended spatiotemporal benchmarks, sequential improvement from kinematics to kinematics + sEMG and then to the fully integrated model, coefficient direction, and later-phase dominance of the integrated neuromechanical signature were consistent across trial-selection methods, sEMG normalization procedures, influential-observation exclusions, and analytical-sample definitions. Sensitivity results are summarized in **Supplementary Table S3**.

## Discussion

This study identified phase-specific neuromechanical signatures of 40-m sprint performance by integrating high-speed two-dimensional sagittal-plane kinematics with synchronized surface electromyography. The principal finding was that sprint performance was characterized more accurately by a coordinated multimodal profile than by kinematic or sEMG information alone. The non-spatiotemporal integrated neuromechanical model improved out-of-fold prediction relative to either single-modality model, while adding neuromechanical information to the direct and extended spatiotemporal benchmarks further reduced prediction error. Importantly, this improvement was not attributable to a single dominant feature. Faster sprinting was associated with a distributed configuration of step mechanics, segment orientation, muscle-excitation magnitude, activation timing, antagonist coordination, and excitation–movement coupling. These findings support the interpretation of sprinting as a dynamically reorganized neuromechanical task rather than a sequence of independent biomechanical variables.

The phase-specific velocity profiles showed that the performance gap between sprint-trained athletes and physically active non-sprinters widened progressively across the sprint. The smallest difference occurred during the initial 10 m, whereas the largest separation emerged during transition and high-speed running. This pattern suggests that the ability to continue reorganizing mechanics and neuromuscular control as velocity rises may distinguish sprint proficiency more strongly than initial acceleration alone (Weyand et al., 2000). Physically active non-sprinters increased velocity during the early intervals but approached a relative plateau after 20 m, while sprint-trained athletes continued to increase speed. The later phases therefore represented not merely faster versions of early acceleration, but mechanically and neuromuscularly distinct performance states requiring shorter support times, more effective limb repositioning, and more precisely timed muscle excitation (Burgomaster et al., 2014; Hunter et al., 2005; Weyand et al., 2000).

The discrete kinematic results demonstrated that trained sprint performance was associated with a progressively more favorable organization of step length, step frequency, and contact–flight structure (Morin et al., 2019; Morin J. B. et al., 2015). Sprint-trained athletes produced longer normalized steps across all phases, while differences in step frequency became more pronounced after the initial acceleration phase (Cappellini et al., 2006). These kinematic characteristics occurred alongside shorter ground-contact times and longer flight times, particularly during transition and high-speed running (Sloniger et al., 1997). The coexistence of longer steps and higher step frequencies indicates that superior performance was not achieved through a simple trade-off between these variables (Chumanov et al., 2011). Instead, sprint-trained athletes displayed greater horizontal displacement per step while preserving rapid step turnover, a pattern consistent with more effective coordination of force application, limb stiffness, and recovery-leg mechanics.

Segment and joint configurations further differentiated the groups. During early acceleration, sprint-trained athletes adopted greater forward trunk inclination, consistent with the mechanical requirement to orient the body and resultant force vector toward horizontal acceleration (Morin J. et al., 2015). As the sprint progressed, they transitioned to a more upright posture than non-sprinters, suggesting a more complete and phase-appropriate reorganization from acceleration mechanics toward high-speed running (Yu et al., 2008). Greater knee extension at initial contact, increased hip extension at toe-off, and greater plantar flexion during later phases were also characteristic of the trained group (Higashihara et al., 2018). These features may facilitate effective limb preparation before ground contact, rapid support-phase force transmission, and continued propulsion despite progressively shorter contact times (Kaltenbach et al., 2024). Because these differences remained after adjustment for phase-specific velocity, they are unlikely to reflect running speed alone and instead indicate a distinctive mechanical organization associated with sprint-training status and performance capability.

The waveform analyses strengthened this interpretation by showing that between-group differences were temporally localized rather than uniformly distributed across stance and swing (Hamner et al., 2010). Differences in trunk orientation and knee configuration were concentrated around initial contact and early stance, whereas hip-extension differences occurred predominantly during late stance. During transition and high-speed running, trained athletes also displayed greater knee flexion during mid-swing followed by more rapid terminal extension (Dorn et al., 2012). This sequence is mechanically meaningful because effective sprinting requires both rapid limb recovery and precise preparation of the foot and lower limb for ground contact. The waveform results therefore added information that could not be obtained from discrete event values alone, indicating that neuromechanical proficiency was associated not only with the magnitude of joint-angle differences but also with their timing within the movement cycle.

The sEMG findings revealed a corresponding shift in neuromuscular emphasis across the sprint. Initial acceleration was distinguished primarily by greater gluteus maximus and vastus lateralis excitation during stance, consistent with the high hip- and knee-extensor demands of overcoming inertia and generating forward acceleration (Yousefian Molla and Fatahi, 2022). These proximal and knee-extensor differences diminished at higher velocities. In contrast, biceps femoris preactivation and medial gastrocnemius stance excitation became increasingly prominent during transition and high-speed running (Clark and Weyand, 2014). This phase-dependent redistribution indicates that trained sprinting is not characterized by globally greater activation, but by selective amplification of the muscles and temporal windows most relevant to the mechanical demands of each phase.

Earlier hamstring excitation before initial contact emerged as one of the most stable late-phase features (Seidl et al., 2021). Greater biceps femoris preactivation may prepare the posterior thigh for the rapid transition from terminal swing to ground contact, contribute to limb deceleration, and increase readiness for force transmission immediately after contact. Similarly, greater medial gastrocnemius excitation during later stance may support ankle stiffness and plantar-flexor contribution when contact duration is minimal (Miyashiro et al., 2019). The finding that sprint-trained athletes exhibited lower biceps femoris–quadriceps and plantar-flexor–dorsiflexor coactivation from continued acceleration onward further suggests that higher performance is associated with selective coordination rather than generalized joint stiffening. Reduced prolonged antagonist coactivation may lower unnecessary internal resistance while preserving the short, task-specific co-contraction required for joint stability and impact preparation.

The excitation–movement coupling results provide an important extension beyond conventional amplitude-based sEMG analysis. Earlier biceps femoris and medial gastrocnemius onset relative to initial contact, peak excitation occurring closer to mid-stance, and plantar-flexor excitation persisting nearer to toe-off indicate tighter temporal alignment between neural activation and mechanical events in trained athletes (Valamatos et al., 2022). These findings suggest that performance-relevant neuromuscular control is partly expressed through timing precision. Two athletes may demonstrate similar average activation amplitudes while differing substantially in whether excitation occurs early enough to prepare the limb, peaks during the mechanically relevant portion of stance, or persists long enough to contribute to propulsion (Haugen and Buchheit, 2016). The integration of event-based kinematics and sEMG therefore revealed dimensions of sprint proficiency that would remain obscured if the modalities were examined independently.

The predictive modelling results represented the central contribution of the study while also distinguishing neuromechanical information from the direct mathematical constituents of running velocity. The direct spatiotemporal benchmark, comprising normalized step length and step frequency, explained 72% of out-of-fold variance ( $R^{2}=0.72$; RMSE = 0.36 m·s^-1^), whereas adding the integrated neuromechanical feature set increased *R*^2^ to 0.86 and reduced RMSE to 0.25 m·s^-1^. The paired RMSE reduction of 0.11 m·s^-1^ (95% CI 0.07–0.15) indicates that the neuromechanical profile provided predictive information beyond the mathematical constituents of velocity. This incremental value persisted when ground-contact and flight times were additionally included in the extended spatiotemporal benchmark, with *R*^2^ increasing from 0.76 to 0.87 and RMSE decreasing from 0.33 to 0.24 m·s^-1^ after addition of the neuromechanical features. The reduction in prediction error relative to both single-modality models demonstrated that mechanical and neuromuscular features contributed complementary information (Haugen and Buchheit, 2016). More specifically, the nested modelling sequence demonstrated that adding sEMG information to non-spatiotemporal kinematics increased cross-validated *R*^2^ from 0.60 to 0.69 and reduced RMSE from 0.43 to 0.38 m·s^-1^, with a paired RMSE reduction of 0.05 m·s^-1^ (95% CI 0.02–0.08). Adding coactivation and excitation–movement coupling variables produced a further increase in *R*^2^ to 0.74 and reduced RMSE to 0.34 m·s^-1^, corresponding to an additional paired reduction of 0.04 m·s^-1^ (95% CI 0.01–0.07). These sequential improvements indicate that sEMG captured performance-relevant information not represented by segmental and joint kinematics alone, while coupling and coordination variables contributed additional information beyond the combination of kinematics and muscle-excitation features. Kinematics described the observable organization of the sprint action, whereas sEMG and coupling variables provided information concerning the neural preparation and coordination underlying that organization (Prampero et al., 2005). Accordingly, the predictive advantage of the integrated profile cannot be attributed solely to mathematical reconstruction of velocity from step length and step frequency. The integrated profile therefore did not simply add more variables; it captured interacting dimensions of performance that were only partially represented within either modality.

The stable non-spatiotemporal signature was dominated by transition- and high-speed-running features, which together accounted for 82% of the summed absolute standardized coefficient magnitude. Biceps femoris preactivation and medial gastrocnemius stance excitation during transition, together with high-speed-running knee configuration, earlier biceps femoris onset, lower biceps femoris–rectus femoris coactivation, and medial gastrocnemius excitation timing, were among the most persistent contributors (Nagahara et al., 2014). These phase-specific percentages represent relative shares of coefficient magnitude rather than independent proportions of explained variance. Nevertheless, the all-phase model remained superior to the high-speed-running-only model. The all-phase integrated neuromechanical model increased cross-validated *R*^2^ from 0.68 to 0.74 and reduced RMSE from 0.38 to 0.34 m·s^-1^ relative to the high-speed-only model. This indicates that total 40-m performance cannot be reduced to maximal-speed mechanics alone (Clark and Ryan, 2022). Early-phase features therefore retained additional predictive information, potentially because initial- and continued-acceleration characteristics are associated with the velocity conditions preceding transition and high-speed running. The final signature should therefore be interpreted as a coordinated sequence of phase-dependent solutions rather than as a single late-sprint phenotype.

The secondary classification analysis reinforced the construct validity of the integrated profile. The integrated model discriminated sprint-trained athletes from physically active non-sprinters with high internally validated accuracy and better calibration than the kinematic-only and sEMG-only models (Morin et al., 2015; Rabita et al., 2015). Seven of the nine stable classification features overlapped with the non-spatiotemporal performance-related signature, indicating substantial but incomplete correspondence between the characteristics distinguishing sprint-trained athletes and those associated with faster sprinting. However, the overlap was incomplete, which is analytically important. Training status and performance are related but not interchangeable outcomes. Unlike the continuous velocity models, the classification analysis could retain spatiotemporal variables because sprint-training status is not mathematically defined by step length, step frequency, contact time, or flight time. Some characteristics may be associated with sprint-specific training exposure without making a proportionally large contribution to current 40-m velocity, whereas other features may predict performance across both groups without serving as strong markers of group membership.

These findings have practical implications for sprint assessment and training design. Athlete profiling based on total sprint time alone cannot identify whether performance limitations arise primarily from early acceleration mechanics, late-phase limb repositioning, insufficient hamstring preactivation, reduced plantar-flexor contribution, or excessive antagonist coactivation. A phase-specific integrated assessment can distinguish these possibilities and support more targeted intervention. In athletes displaying adequate early extensor excitation but limited late-phase performance, maximal-velocity mechanics, posterior-chain preparation, ankle stiffness, and terminal-swing control may represent relevant targets for further assessment and intervention testing. Conversely, in athletes with strong late-phase neuromuscular characteristics but weak initial acceleration, projection mechanics and proximal extensor force application may represent candidate targets for future intervention studies. The present framework also demonstrates that high-speed 2D video combined with wireless sEMG can provide a practical field-based approach to neuromechanical profiling without requiring a fully instrumented laboratory.

Several methodological features strengthen the interpretation of the findings, including balanced reference groups, phase-specific velocity adjustment, synchronized multimodal acquisition, waveform-level analysis, formal reliability screening, separation of direct and proximal spatiotemporal variables from the primary neuromechanical performance signature, and nested internal validation. The sequential comparison of kinematics, kinematics + sEMG, and the fully integrated model further allowed the incremental predictive information associated with muscle excitation and neuromechanical coupling to be quantified under identical cross-validation folds. The prespecified candidate-feature inventory and transparent selection pipeline, encompassing reliability screening, fold-specific correlation reduction, elastic-net regularization, and stability assessment, further strengthened analytical reproducibility while minimizing arbitrary feature retention and information leakage from the validation data. Although the initial non-spatiotemporal feature space was high-dimensional relative to the complete multimodal sample, the effective complexity of the fitted models was substantially reduced by correlation filtering and regularization, with a median of 22 non-zero coefficients per internal-validation resample and only nine features satisfying the final stability criterion. Accordingly, the present sample should be interpreted as supporting internally validated regularized model development rather than unrestricted estimation of the full candidate feature set, with repeated nested cross-validation used to limit optimism in model performance under this feature-to-sample ratio. The study nevertheless examined sagittal-plane behavior within a cross-sectional design, and the derived signatures should therefore be interpreted as performance-associated profiles rather than causal training adaptations. The use of surface electromyography also limits inference to the recorded superficial muscles. A further limitation is that the predictive models were not externally validated in an independent cohort. Although repeated nested cross-validation, regularization, stability selection, sensitivity analyses, and benchmark comparisons were used to reduce optimism within the present sample, these procedures do not substitute for external validation. The relatively high initial predictor-to-participant ratio further reinforces the need for independent replication, and the transportability, calibration, predictive performance, and feature stability of the identified neuromechanical signature should therefore be confirmed in a larger external cohort. These considerations do not alter the primary conclusion but indicate that external validation and longitudinal testing will be valuable for determining how responsive the identified signatures are to targeted training.

## Conclusion

This study demonstrates that 40-m sprint performance is characterized by a phase-specific neuromechanical signature integrating segment orientation, joint configuration, muscle-excitation magnitude, activation timing, antagonist coordination, and excitation–movement coupling. Early acceleration was distinguished primarily by forward-oriented posture and greater proximal extensor excitation, whereas transition and high-speed running were characterized by greater hamstring preactivation, stronger plantar-flexor excitation, distinct knee and trunk configurations, earlier pre-contact activation, and reduced antagonist coactivation. Importantly, the integrated neuromechanical features provided incremental predictive information beyond the direct spatiotemporal constituents of running velocity, with this advantage persisting when ground-contact and flight times were additionally considered. The integrated kinematic–sEMG model provided greater internally validated predictive and discriminative performance than either modality alone, indicating that sprint proficiency cannot be adequately represented by isolated biomechanical or neuromuscular variables. These findings support an integrated, phase-specific framework for field-based sprint profiling and targeted performance assessment, while external validation is required before broader generalization.

## Data Availability

The original contributions presented in the study are included in the article/**Supplementary Material**. Further inquiries can be directed to the corresponding author.
