# Supplementary material for: Phase-specific neuromechanical signatures of 40-m sprint performance: an integrated two-dimensional kinematic and surface electromyographic profiling study
Source: Front Physiol. 2026 Oct 9;17:1939386. doi: 10.3389/fphys.2026.1939386 (PMC13690675; doi:10.3389/fphys.2026.1939386)
Supplement: Supplementary file 1 [file Table1.docx]

**Supplementary Figure S1.**

**Supplementary Figure S1.** Participant flow and modality-specific data availability. Timing, kinematic, sEMG, and integrated analyses included 160, 158, 154, and 152 participants, respectively. sEMG, surface electromyography.

**Supplementary Table S2**

**Complete adjusted phase-specific discrete kinematic outcomes according to sprint-training status**

Panel A. Spatiotemporal outcomes

| **Outcome** | **Initial acceleration (0–10 m)** | **Continued acceleration (10–20 m)** | **Transition (20–30 m)** | **High-speed running (30–40 m)** | **Group (p)** | **Phase (p)** | **Group × phase (p)** |
| --- | --- | --- | --- | --- | --- | --- | --- |
| Normalized step length, leg lengths | 1.38 ± 0.03 / 1.30 ± 0.03; Δ 0.08 (0.03 to 0.13), (p=.002) | 1.83 ± 0.03 / 1.69 ± 0.03; Δ 0.14 (0.08 to 0.20), (p<.001) | 2.03 ± 0.04 / 1.82 ± 0.04; Δ 0.21 (0.14 to 0.28), (p<.001) | 2.13 ± 0.04 / 1.87 ± 0.04; Δ 0.26 (0.19 to 0.33), (p<.001) | <.001 | <.001 | <.001 |
| Step frequency, Hz | 3.73 ± 0.05 / 3.68 ± 0.05; Δ 0.05 (−0.04 to 0.14), (p=.286) | 4.22 ± 0.06 / 4.04 ± 0.06; Δ 0.18 (0.07 to 0.29), (p=.001) | 4.45 ± 0.06 / 4.19 ± 0.06; Δ 0.26 (0.14 to 0.38), (p<.001) | 4.53 ± 0.06 / 4.21 ± 0.06; Δ 0.32 (0.20 to 0.44), (p<.001) | <.001 | <.001 | <.001 |
| Ground-contact time, s | 0.171 ± 0.003 / 0.184 ± 0.003; Δ −0.013 (−0.019 to −0.007), (p<.001) | 0.136 ± 0.003 / 0.153 ± 0.003; Δ −0.017 (−0.023 to −0.011), (p<.001) | 0.116 ± 0.004 / 0.139 ± 0.004; Δ −0.023 (−0.030 to −0.016), (p<.001) | 0.107 ± 0.004 / 0.131 ± 0.004; Δ −0.024 (−0.031 to −0.017), (p<.001) | <.001 | <.001 | <.001 |
| Flight time, s | 0.071 ± 0.003 / 0.064 ± 0.003; Δ 0.007 (0.001 to 0.013), (p=.018) | 0.091 ± 0.004 / 0.079 ± 0.004; Δ 0.012 (0.005 to 0.019), (p=.001) | 0.108 ± 0.004 / 0.089 ± 0.004; Δ 0.019 (0.011 to 0.027), (p<.001) | 0.116 ± 0.004 / 0.095 ± 0.004; Δ 0.021 (0.013 to 0.029), (p<.001) | <.001 | <.001 | <.001 |

Panel B. Trunk and hip kinematics

| **Outcome** | **Initial acceleration (0–10 m)** | **Continued acceleration (10–20 m)** | **Transition (20–30 m)** | **High-speed running (30–40 m)** | **Group (p)** | **Phase (p)** | **Group × phase (p)** |
| --- | --- | --- | --- | --- | --- | --- | --- |
| Trunk inclination at initial contact, ° | 43.2 ± 1.1 / 39.0 ± 1.1; Δ 4.2 (1.6 to 6.8), (p=.002) | 31.1 ± 1.2 / 27.3 ± 1.2; Δ 3.8 (1.1 to 6.5), (p=.006) | 20.1 ± 1.3 / 23.2 ± 1.3; Δ −3.1 (−5.9 to −0.3), (p=.029) | 14.2 ± 1.3 / 19.6 ± 1.3; Δ −5.4 (−8.2 to −2.6), (p<.001) | .417 | <.001 | <.001 |
| Trunk inclination at mid-stance, ° | 39.0 ± 1.0 / 35.8 ± 1.0; Δ 3.2 (0.9 to 5.5), (p=.007) | 26.4 ± 1.1 / 24.1 ± 1.1; Δ 2.3 (−0.2 to 4.8), (p=.078) | 16.3 ± 1.2 / 18.2 ± 1.2; Δ −1.9 (−4.5 to 0.7), (p=.153) | 10.8 ± 1.3 / 15.1 ± 1.3; Δ −4.3 (−7.1 to −1.5), (p=.003) | .268 | <.001 | .002 |
| Trunk inclination at toe-off, ° | 35.8 ± 1.0 / 33.2 ± 1.0; Δ 2.6 (0.4 to 4.8), (p=.024) | 22.0 ± 1.1 / 20.7 ± 1.1; Δ 1.3 (−1.1 to 3.7), (p=.286) | 12.8 ± 1.2 / 14.1 ± 1.2; Δ −1.3 (−3.9 to 1.3), (p=.326) | 8.2 ± 1.3 / 11.8 ± 1.3; Δ −3.6 (−6.4 to −0.8), (p=.012) | .482 | <.001 | .018 |
| Trunk stance-phase range of motion, ° | 7.4 ± 0.5 / 5.8 ± 0.5; Δ 1.6 (0.5 to 2.7), (p=.005) | 9.1 ± 0.6 / 6.6 ± 0.6; Δ 2.5 (1.2 to 3.8), (p<.001) | 7.3 ± 0.7 / 9.1 ± 0.7; Δ −1.8 (−3.3 to −0.3), (p=.017) | 6.0 ± 0.7 / 7.8 ± 0.7; Δ −1.8 (−3.4 to −0.2), (p=.027) | .613 | <.001 | .006 |
| Hip flexion at initial contact, ° | 56.2 ± 1.4 / 53.9 ± 1.4; Δ 2.3 (−0.8 to 5.4), (p=.145) | 49.1 ± 1.5 / 47.0 ± 1.5; Δ 2.1 (−1.2 to 5.4), (p=.209) | 42.8 ± 1.6 / 41.2 ± 1.6; Δ 1.6 (−1.9 to 5.1), (p=.368) | 39.0 ± 1.7 / 37.8 ± 1.7; Δ 1.2 (−2.5 to 4.9), (p=.524) | .143 | <.001 | .681 |
| Hip flexion at mid-stance, ° | 31.2 ± 1.2 / 30.5 ± 1.2; Δ 0.7 (−1.9 to 3.3), (p=.598) | 22.4 ± 1.3 / 22.0 ± 1.3; Δ 0.4 (−2.4 to 3.2), (p=.779) | 15.1 ± 1.4 / 15.8 ± 1.4; Δ −0.7 (−3.7 to 2.3), (p=.645) | 11.0 ± 1.5 / 12.1 ± 1.5; Δ −1.1 (−4.3 to 2.1), (p=.501) | .774 | <.001 | .742 |
| Hip extension at toe-off, ° | 9.3 ± 0.9 / 7.2 ± 0.9; Δ 2.1 (0.2 to 4.0), (p=.031) | 13.2 ± 1.0 / 9.2 ± 1.0; Δ 4.0 (1.8 to 6.2), (p<.001) | 17.1 ± 1.1 / 11.0 ± 1.1; Δ 6.1 (3.6 to 8.6), (p<.001) | 19.0 ± 1.2 / 12.0 ± 1.2; Δ 7.0 (4.4 to 9.6), (p<.001) | <.001 | <.001 | <.001 |
| Hip stance-phase range of motion, ° | 65.5 ± 1.5 / 61.1 ± 1.5; Δ 4.4 (1.1 to 7.7), (p=.009) | 62.3 ± 1.6 / 56.2 ± 1.6; Δ 6.1 (2.5 to 9.7), (p=.001) | 59.9 ± 1.8 / 52.2 ± 1.8; Δ 7.7 (3.8 to 11.6), (p<.001) | 58.0 ± 1.9 / 49.8 ± 1.9; Δ 8.2 (4.0 to 12.4), (p<.001) | <.001 | <.001 | .032 |

Panel C. Knee and ankle kinematics

| **Outcome** | **Initial acceleration (0–10 m)** | **Continued acceleration (10–20 m)** | **Transition (20–30 m)** | **High-speed running (30–40 m)** | **Group (p)** | **Phase (p)** | **Group × phase (p)** |
| --- | --- | --- | --- | --- | --- | --- | --- |
| Knee angle at initial contact, ° | 150.1 ± 1.3 / 145.3 ± 1.3; Δ 4.8 (1.9 to 7.7), (p=.001) | 156.4 ± 1.5 / 149.7 ± 1.5; Δ 6.7 (3.4 to 10.0), (p<.001) | 162.0 ± 1.7 / 153.1 ± 1.7; Δ 8.9 (5.2 to 12.6), (p<.001) | 166.3 ± 1.8 / 155.6 ± 1.8; Δ 10.7 (6.9 to 14.5), (p<.001) | <.001 | <.001 | <.001 |
| Knee angle at mid-stance, ° | 129.4 ± 1.6 / 127.8 ± 1.6; Δ 1.6 (−1.9 to 5.1), (p=.372) | 137.3 ± 1.7 / 134.9 ± 1.7; Δ 2.4 (−1.4 to 6.2), (p=.216) | 143.1 ± 1.9 / 140.8 ± 1.9; Δ 2.3 (−1.8 to 6.4), (p=.270) | 147.0 ± 2.0 / 144.1 ± 2.0; Δ 2.9 (−1.5 to 7.3), (p=.194) | .182 | <.001 | .644 |
| Knee angle at toe-off, ° | 166.2 ± 1.2 / 163.9 ± 1.2; Δ 2.3 (−0.4 to 5.0), (p=.094) | 170.4 ± 1.3 / 166.9 ± 1.3; Δ 3.5 (0.6 to 6.4), (p=.018) | 173.0 ± 1.5 / 168.6 ± 1.5; Δ 4.4 (1.2 to 7.6), (p=.007) | 175.1 ± 1.6 / 169.5 ± 1.6; Δ 5.6 (2.2 to 9.0), (p=.001) | <.001 | <.001 | .009 |
| Knee stance-phase range of motion, ° | 31.5 ± 1.4 / 29.8 ± 1.4; Δ 1.7 (−1.3 to 4.7), (p=.267) | 27.8 ± 1.5 / 25.6 ± 1.5; Δ 2.2 (−1.0 to 5.4), (p=.178) | 23.1 ± 1.6 / 20.4 ± 1.6; Δ 2.7 (−0.7 to 6.1), (p=.121) | 19.6 ± 1.7 / 16.7 ± 1.7; Δ 2.9 (−0.8 to 6.6), (p=.126) | .121 | <.001 | .438 |
| Ankle plantar flexion at initial contact, ° | 3.2 ± 1.1 / 0.2 ± 1.1; Δ 3.0 (0.5 to 5.5), (p=.019) | 6.4 ± 1.2 / 2.1 ± 1.2; Δ 4.3 (1.6 to 7.0), (p=.002) | 9.1 ± 1.3 / 3.7 ± 1.3; Δ 5.4 (2.5 to 8.3), (p<.001) | 12.0 ± 1.4 / 5.2 ± 1.4; Δ 6.8 (3.8 to 9.8), (p<.001) | <.001 | <.001 | <.001 |
| Ankle dorsiflexion at mid-stance, ° | 12.8 ± 1.1 / 14.0 ± 1.1; Δ −1.2 (−3.7 to 1.3), (p=.344) | 10.6 ± 1.2 / 12.9 ± 1.2; Δ −2.3 (−5.0 to 0.4), (p=.094) | 8.4 ± 1.3 / 11.8 ± 1.3; Δ −3.4 (−6.3 to −0.5), (p=.022) | 7.1 ± 1.4 / 11.1 ± 1.4; Δ −4.0 (−7.0 to −1.0), (p=.009) | .009 | <.001 | .028 |
| Ankle plantar flexion at toe-off, ° | 23.5 ± 1.4 / 21.8 ± 1.4; Δ 1.7 (−1.4 to 4.8), (p=.282) | 27.9 ± 1.5 / 24.7 ± 1.5; Δ 3.2 (−0.2 to 6.6), (p=.066) | 31.4 ± 1.6 / 27.0 ± 1.6; Δ 4.4 (0.8 to 8.0), (p=.017) | 34.1 ± 1.7 / 28.8 ± 1.7; Δ 5.3 (1.5 to 9.1), (p=.006) | .002 | <.001 | .063 |
| Ankle stance-phase range of motion, ° | 36.3 ± 1.8 / 35.8 ± 1.8; Δ 0.5 (−3.4 to 4.4), (p=.802) | 38.5 ± 1.9 / 37.6 ± 1.9; Δ 0.9 (−3.3 to 5.1), (p=.675) | 39.8 ± 2.0 / 38.8 ± 2.0; Δ 1.0 (−3.5 to 5.5), (p=.661) | 41.2 ± 2.2 / 39.9 ± 2.2; Δ 1.3 (−3.5 to 6.1), (p=.595) | .594 | <.001 | .427 |

**Supplementary Table S3. Sensitivity and robustness analyses of the performance-related and classification models**

**Panel A.** Robustness of the benchmark and augmented performance models

| Analytical condition | Sample, $n$ | Direct benchmark $R^{2}$ | Direct benchmark RMSE | Augmented direct $R^{2}$ | Augmented direct RMSE | Extended benchmark $R^{2}$ | Extended benchmark RMSE | Augmented extended $R^{2}$ | Augmented extended RMSE | Stable features retained |
| --- | --- | --- | --- | --- | --- | --- | --- | --- | --- | --- |
| Primary analysis: fastest valid trial | 152 | 0.72 | 0.36 | 0.86 | 0.25 | 0.76 | 0.33 | 0.87 | 0.24 | 9/9 |
| Mean of two fastest trials | 148 | 0.70 | 0.37 | 0.84 | 0.26 | 0.74 | 0.34 | 0.85 | 0.25 | 8/9 |
| Sprint-derived sEMG normalization | 152 | 0.72 | 0.36 | 0.85 | 0.26 | 0.76 | 0.33 | 0.86 | 0.25 | 8/9 |
| Influential observations excluded | 147 | 0.73 | 0.35 | 0.87 | 0.24 | 0.77 | 0.32 | 0.88 | 0.23 | 9/9 |

**Panel B.** Robustness to analytical sample and sEMG normalization

| Analytical condition | Sample, $n$ | Cross-validated $R^{2}$ | RMSE, m·s${}^{-1}$ | MAE, m·s${}^{-1}$ | Classification AUC | Balanced accuracy |
| --- | --- | --- | --- | --- | --- | --- |
| Integrated neuromechanical, primary analysis | 152 | 0.74 | 0.34 | 0.26 | 0.94 | 0.89 |
| Integrated neuromechanical, sprint-derived sEMG normalization | 152 | 0.73 | 0.35 | 0.27 | 0.93 | 0.88 |
| Non-spatiotemporal kinematic, complete-case sample | 152 | 0.60 | 0.43 | 0.34 | — | — |
| Non-spatiotemporal kinematic, full sample | 158 | 0.61 | 0.42 | 0.33 | — | — |
| sEMG-only, complete-case sample | 152 | 0.55 | 0.45 | 0.35 | 0.84 | 0.78 |
| sEMG-only, full sample | 154 | 0.56 | 0.44 | 0.34 | 0.85 | 0.79 |

**Panel C.** Incremental contribution of sEMG and coupling variables beyond non-spatiotemporal kinematics

| Analytical condition | $n$ | Kinematics $R^{2}$ | Kinematics RMSE | Kinematics + sEMG $R^{2}$ | Kinematics + sEMG RMSE | Integrated $R^{2}$ | Integrated RMSE | RMSE reduction after adding sEMG | RMSE reduction after adding coupling |
| --- | --- | --- | --- | --- | --- | --- | --- | --- | --- |
| Primary analysis: fastest valid trial | 152 | 0.60 | 0.43 | 0.69 | 0.38 | 0.74 | 0.34 | 0.05 (0.02–0.08) | 0.04 (0.01–0.07) |
| Mean of two fastest trials | 148 | 0.58 | 0.44 | 0.67 | 0.39 | 0.72 | 0.36 | 0.05 (0.02–0.08) | 0.03 (0.01–0.06) |
| Sprint-derived sEMG normalization | 152 | 0.60 | 0.43 | 0.68 | 0.39 | 0.73 | 0.35 | 0.04 (0.01–0.07) | 0.04 (0.01–0.07) |
| Influential observations excluded | 147 | 0.62 | 0.42 | 0.70 | 0.37 | 0.75 | 0.33 | 0.05 (0.02–0.08) | 0.04 (0.01–0.07) |

**Supplementary Table S4. Candidate feature pool and feature-selection pipeline for neuromechanical signature development**

**Panel A.** Composition of the prespecified candidate feature pool

| Feature domain | Feature construction | Phase-specific feature count | Initial candidate features | Excluded by reliability screening | Reliability-qualified features | Role in primary continuous-velocity modelling |
| --- | --- | --- | --- | --- | --- | --- |
| Spatiotemporal | Normalized step length, step frequency, ground-contact time, and flight time across four sprint phases | 4 × 4 | 16 | 0 | 16 | Separated from primary neuromechanical pool; used in direct and extended benchmark models |
| Non-spatiotemporal kinematics | Trunk, hip, knee, and ankle configurations at prespecified events and stance-phase excursion across four sprint phases | 16 × 4 | 64 | 0 | 64 | Eligible for kinematic and integrated neuromechanical models |
| sEMG amplitude and timing | Phase-specific excitation amplitude and timing summaries from six muscles | 42 × 4 | 168 | 2 | 166 | Eligible for sEMG-only, kinematics + sEMG, and integrated models |
| Coactivation | Biceps femoris–rectus femoris, biceps femoris–vastus lateralis, and medial gastrocnemius–tibialis anterior indices across four phases | 3 × 4 | 12 | 0 | 12 | Added only in the fully integrated model |
| Excitation–movement coupling | Excitation onset–initial contact, peak excitation–mid-stance, and excitation offset–toe-off for six muscles across four phases | 18 × 4 | 72 | 0 | 72 | Added only in the fully integrated model |
| Total |  |  | 332 | 2 | 330 |  |

**Panel B.** Sequential feature-selection pipeline for the primary performance-related signature

| Selection stage | Procedure | Number of features remaining |
| --- | --- | --- |
| Prespecified initial candidate pool | All phase-specific discrete spatiotemporal, kinematic, sEMG, coactivation, and excitation–movement coupling features | 332 |
| Reliability screening | Features required ICC ≥ 0.75 and coefficient of variation ≤20% | 330 |
| Separation of spatiotemporal variables | Sixteen phase-specific step length, step frequency, contact-time, and flight-time features reserved for benchmark models | 314 |
| Training-fold standardization | Continuous predictors standardized using parameters estimated only from the corresponding training data | 314 |
| Fold-specific correlation filtering | Predictors with ( | r |
| Elastic-net regularization | Predictors with non-zero coefficients within individual internal-validation resamples | Median 22 (IQR 18–26) |
| Stability screening | Non-zero coefficient in ≥70% of resamples plus consistent coefficient direction | 9 |
| Final primary performance-related signature | Stable non-spatiotemporal neuromechanical features retained for interpretation | 9 |

**Panel C.** Candidate pool size according to predictive model

| Predictive model | Eligible feature domains | Reliability-qualified features before fold-specific correlation filtering | Median retained after correlation filtering (range) |
| --- | --- | --- | --- |
| Non-spatiotemporal kinematic | Segmental and joint kinematics | 64 | 38 (35–41) |
| sEMG only | sEMG amplitude and excitation/timing features | 166 | 71 (65–77) |
| Kinematics + sEMG | Non-spatiotemporal kinematics + sEMG | 230 | 96 (89–103) |
| Fully integrated neuromechanical | Kinematics + sEMG + coactivation + excitation–movement coupling | 314 | 121 (112–131) |
| Direct spatiotemporal benchmark | Normalized step length + step frequency across four phases | 8 | Not applicable |
| Extended spatiotemporal benchmark | Direct benchmark + ground-contact time + flight time across four phases | 16 | Not applicable |
| Augmented direct benchmark | Fully integrated neuromechanical pool + direct benchmark | 322 | Fold-specific |
| Augmented extended benchmark | Fully integrated neuromechanical pool + extended benchmark | 330 | Fold-specific |
| Sprint-training-status classification | Full reliability-qualified feature pool, including spatiotemporal features | 330 | Fold-specific |
